# Comparison of nutritional composition between plant-based drinks and cow’s milk

**DOI:** 10.3389/fnut.2022.988707

**Published:** 2022-10-28

**Authors:** Barbara Walther, Dominik Guggisberg, René Badertscher, Lotti Egger, Reto Portmann, Sébastien Dubois, Max Haldimann, Katrin Kopf-Bolanz, Peter Rhyn, Otmar Zoller, Rosmarie Veraguth, Serge Rezzi

**Affiliations:** ^1^Agroscope, Bern, Switzerland; ^2^Risk Assessment Division, Federal Food Safety and Veterinary Office, Bern, Switzerland; ^3^School of Agricultural, Forestry and Food Sciences, Bern University of Applied Sciences, Zollikofen, Switzerland; ^4^Swiss Nutrition and Health Foundation, Épalinges, Switzerland

**Keywords:** plant-based drink, cow’s milk, nutritional composition, nutrient analysis, residue, RDA

## Abstract

The high decline in liquid milk consumption in Western countries has been compensated by the increased consumption of processed dairy products and the rapidly increasing number of new plant-based beverages constantly introduced in the market, advertised as milk substitutes and placed on shelves near milk products. To provide better understanding about the nutritional value of these drinks compared with cow’s milk, 27 plant-based drinks of 8 different species and two milk samples were purchased from two big retailers in Switzerland, and their composition regarding protein, carbohydrate, fat, vitamin, and mineral contents and residue load [glyphosate, aminomethylphosphonic acid (AMPA), and arsenic] was analyzed quantitatively and qualitatively. Energy and nutrient intakes were calculated and compared with the dietary reference values for Germany, Austria and Switzerland (D-A-CH). In addition, the digestible indispensable amino acid score (DIAAS) was calculated to estimate the quality of the proteins. Milk contained more energy; fat; carbohydrate; vitamins C, B_2_, B_12_, and A; biotin; pantothenic acid; calcium; phosphorus; and iodine than most plant-based drinks. Soy drinks provided slightly more protein and markedly more vitamins B_1_ and B_6_, folic acid, and vitamins E and D_2_ (with supplemented vitamin D_2_) and K_1_, magnesium, manganese, iron, and copper than milk and the other plant-based drinks. However, with the exception of cow’s milk and soy drinks, which had > 3% protein, most milk alternatives contained ≤ 1% protein; therefore, they cannot be considered good protein sources. In regard to protein quality, milk was outstanding compared with all plant-based drinks and exhibited higher calculated DIAASs. Our results show that the analyzed plant-based drinks are not real alternatives to milk in terms of nutrient composition, even if the actual fortification is taken into account. Improved fortification is still an issue and can be optimized using the most bioavailable and soluble derivatives. Complete replacement of milk with plant-based drinks without adjusting the overall diet can lead to deficiencies of certain important nutrients in the long term.

## Introduction

The dairy milk consumption by humans dates back to *circa* 7000 BC, when a modification of the gene responsible for the lactase production prevented the progressive loss of lactase activity in early childhood following weaning, leading to a lactose-persistent adult phenotype. Owing to this ability to effectively digest lactose throughout adulthood, dairy milk offered an evolutionary advantage due to its hydration and rich nutritional content (1). Today, dairy milk and dairy product consumptions are promoted in public health policies worldwide and considered to play a crucial role in human nutrition (2).

The worldwide milk consumption is increasing but this is mainly due to growth in developing countries (3). Conversely, in Switzerland, similarly to most developed countries, consumption of “liquid milk” had been dramatically declining, from 233 L per capita in 1950 to 51 L per capita in 2020 (4). The Swiss National Nutrition Survey “menuCH” reported a dairy intake of two portions per day in the Swiss population as opposed to the three daily portions recommended in Switzerland (5). Several reasons may explain these changes in nutritional behavior. On one hand is a trend to replace fresh milk with more extensively processed dairy products like sweetened milk drinks or fermented milk like yogurt, sour milk, or cheese (4). On the other hand, discussions on sustainability and carbon footprint have led to criticisms of the environmental impacts of animal products, encouraging change toward a more plant-based diet in the general population, not only in strict vegan consumers (6). In addition, with the increasing accessibility of genetic testing (1), the awareness of the prevalence of lactose intolerance, which often leads to reduced traditional dairy consumption despite the availability of lactose-free dairy products and the substitution of dairy milk with plant-based drinks, has been increasing (7). Plant-based protein sources can provide alternatives to milk for people who are allergic to milk protein. However, this assumption must first be tested (8).

More recently, the consumption of plant-based substitutes for dairy milk has increased (9). Furthermore, with the expansion of consumer interest in the plant-based alternative market, not only the non-dairy industry but also, recently, the dairy industry has started to extensively develop and promote a wide range of novel plant-based drinks, driving even further the consumer market for these products. Plant-based alternatives initially based almost exclusively on soy and almond includes solutions prepared from rice, oat, nuts, and legumes (10). In the context of this changing market, the average consumer profile is changing from one requiring an exclusively dairy-free diet (e.g., due to food allergy or a vegan diet) to one that may temporarily, intermittently, or permanently replace milk with these alternatives (11).

It is widely accepted that animal and plant foods differ in composition. Proteins of plant origin do not have the same nutritional qualities as those of animal origin. Major differences besides protein content exist between naturally occurring nutrients in dairy milk and plant-based alternative drinks, such as vitamin B_12_, calcium, fiber, and fat compositions and concentrations. Differences in nutrient profile between plant-based drinks and milk have been systematically compared in earlier studies (12–17). However, most of these studies were limited to the evaluation of a few nutrients such as proteins, fats, carbohydrates, and selected vitamins and minerals. In the abovementioned studies, the information was also mostly based on nutrient labeling rather than on actual nutrient determination in the investigated products. Furthermore, with the rapidly expanding market of distinct plant-based alternative drinks with novel formulations, it is important to review and compare the nutritional compositions of the wide range of currently available products to understand their potential impacts on consumer nutrient supply.

The aim of our study was to investigate the comprehensive spectrum of macro-and micro-nutrients in various plant-based drinks sold in the Swiss market and to compare the values with those in full fat cow’s milk, called “milk” in the sequel. When multiple products are available per category, variability within the category was also of interest.

Full fat milk was chosen to also compare fat quality. Moreover, on the basis of the recommended dietary allowance (RDA) values, we calculated the effects of complete substitution of milk with plant-based drinks on the consumer nutrient supply to support regulatory authorities in defining and revising nutritional recommendations for vegans or people refraining completely from milk consumption.

## Materials and methods

### Samples

A total of 36 plant-based products and two samples of cow’s milk were collected from two major supermarkets in Bern, Switzerland, and the surrounding area in August 2019. Only varieties based on one raw ingredient were finally selected, and drinks containing mixtures of raw ingredients were excluded. After exclusion of the mixtures, 27 products from 8 categories remained. Depending on category, 1 to 7 different samples were available on the market. The selected drinks were compared with UHT bovine whole milk with 3.5% fat. The samples were kept at room temperature (for measurements requiring freeze-drying) or directly frozen at −20°C prior to analysis. All drinks were analyzed individually in duplicates or triplicates depending on analysis. Supplementary Table 1 displays an overview of the products and the ingredients listed on their packaging.

### Assessments of sample nutrient composition

The energy values were calculated from the three macronutrients and compared to the information on the label. For calculation the usual values for protein (4 kcal g^–1^), carbohydrates (4 kcal g^–1^), fat (9 kcal g^–1^) are used. Sample dry mass was determined gravimetrically. The drying method using a hot air oven at 102 ± 2°C is the most commonly used method for determining total solids content in food laboratories. The method used is based on the reference method ISO 6731 (18). Instead of pre-drying on a boiling water bath, a test portion was mixed with dried silica sand and then dried in a drying oven at a temperature of 102°C ± 2°C.

### Macronutrient composition measurements

The total protein content was determined for all samples using a copper sulfate/titanium dioxide catalyst, in accordance with the Kjeldahl principle. For the calculation of crude protein out of the determined nitrogen concentration, a conversion factor of 6.38 was used for milk and 5.6 for plant-based products (19). Nitrogen Conversion Factor depends on amino acid composition and this composition is extremely dependent on plant variety, plant parts used, etc. Therefore, a general factor of 5.6 was used, knowing that this factor can only reflect an estimate of the effective protein content. The total amino acid content after acidic hydrolysis was measured using ultra performance liquid chromatography (UPLC) analysis, in accordance with the method of Jaudzems et al. (20). Briefly, the samples were incubated at 110°C in 6 mol L^–1^ HCl for 15 h. The solutions were neutralized and derivatized with an AccQ-Tag Ultra reagent (Waters, Baden-Dätwil, Switzerland), and the amino acid profile was analyzed with ultra-high-performance liquid chromatography (2.1 × 100 mm, 1.7 μm; Acquity UPLC BEH C18, Waters) coupled with a UV detector (Ultimate 3000 RS, Thermo Scientific, Reinach, Switzerland). Tryptophan was measured after alkaline hydrolysis. For this purpose, 500 μl of the sample in 4 ml preparations [6 mol/l NaOH, 16 μg/ml 1-methyl-tryptophan (internal standard), 40 mg/ml starch] were gassed with N2 and incubated at 110°C for 20 h. After cooling the samples to room temperature, they were mixed with 5 ml phosphate buffer (0.2 M, pH7). 100 μl of this was added to 50 μl ice-cold 6 M HCl for neutralization and made up to 2.5 ml with phosphate buffer (0.2 M, pH7). 200 μl were filtered through cellulose and measured by UPLC (column: Waters, Acquity UPLC BEH C18, 1.7 μm, 2.1 × 150 mm, mobile phase: AccQ-Tag Buffer A with 0.1% AccQ-Tag Buffer B, gradient: isocratic, detection: fluorescence extinction 340 nm and emission 360 nm). The amounts were normalized with an external test solution using the internal standard. Digestible indispensable amino acid scores (DIAASs) were calculated on the basis of the assumption that proteins are fully digestible (i.e., 100% digestibility). The amount of indispensable amino acids per gram of food protein was compared with the values provided by the Food and Agriculture Organization (21) for different age groups, resulting in the percentage of food protein that covers the required amounts of indispensable amino acids per gram of food proteins. For legal purposes, the values for growing children have to be used.

Sample total fat content was determined gravimetrically using the method of Weibull-Stoldt (22). After hydrolysis of the proteins and carbohydrates with hydrochloric acid, fat was extracted from the residue with ether in a Soxhlet apparatus. The extracting agent was evaporated, and the fat residue was weighed back. The analytical method for high-resolution lipid analysis with GC-FID is described elsewhere (23). Briefly, to determine the fatty acid distribution, fat extracted from the sample material was dissolved in hexane and transesterified with a methanolic potassium hydroxide solution. The resulting methyl esters were then separated on a gas chromatograph and measured with a flame ionization detector.

The lactose, saccharose, fructose, and glucose contents of the samples were analyzed with a UV-Vis automate (Gallery Analyzer, Thermo, Switzerland), using adapted enzymatic methods based on commercial kits (lactose: E8130, glucose: E8140, saccharose: E8180; R-Biopharm, Switzerland). The starch contents of the samples were measured with an enzymatic assay kit (Total Starch Assay Kit, K-TSTA-50A, Megazyme, Ireland).

### Measurements of micronutrient and trace element composition

#### Vitamins

In all samples, water-soluble vitamin C; biotin; niacin; pantothenic acid; vitamins B_1_, B_2_, B_6_, and B_12_; folic acid; and the fat-soluble vitamins A, E, D_2_, K_1_, and K_2_ were analyzed. Before the vitamin content analyses, all samples were freeze-dried (Christ Epsilon 2–25 D, Martin Christ Gefriertrocknungsanlagen GmbH, Osterode am Harz, Germany) and subsequently ground. The amounts of vitamins B_1_ (24), B_2_ (940.33) (25), and B_6_ (961.15) (25); niacin (944.13) (25); pantothenic acid (945.74) (25); biotin (26); folic acid (27); and vitamin B_12_ (952.20) (25) were analyzed using a microbiological method in 96-well microplates and then measured with turbidimetry (Microplate Spectrophotometer, Epoch, BioTek Instruments, Agilent Technologies, Santa Clara, CA, USA) (Supplementary Table 2). The amounts of vitamins C (28), A (2001.13) (25), and E (992.03) (25), and the carotenoids (29) were measured using HPLC (Flexar, Perkin Elmer, USA) equipped with C18 columns (Macherey Nagel, D and Suplex PKB, Supelco) and UV detection. The amounts of the vitamin D forms (2002.05) (25) were measured with HPLC (Flexar, Perkin Elmer, USA) equipped with C18 column (Macherey Nagel, D) and UV detection, and those of vitamins K_1_ and K_2_ (30) were measured with HPLC followed by a fluorometric detection step (FL 3000, Spectra-system, Thermo). Further details are described in Supplementary Table 2 in the Supplementary material.

#### Minerals

In all samples, the phosphorus, sodium, manganese, magnesium, potassium, iron, copper, calcium, zinc, selenium, sulfur, iodine, chloride, and ash contents were measured. Before the mineral content analyses (P, Na, Mn, Mg, K, Fe, Cu, Ca, and Zn), all samples were freeze-dried (Christ Delta 2–24, Kühner AG, Birsfelden, Switzerland) and subsequently milled (Grindomix knife mill, Restch GmbH, Switzerland).

The mineral contents of the samples were analyzed in accordance with EN 15510:2008 by ICP-OES (ICP-OES Optima 7300, Perkin Elmer, Schwarzenbach, Switzerland) after microwave digestion. The samples were dissolved in a glass tube (5 mL of HNO_3_ 65% + 3 mL of H_2_O ASTM class I) using a microwave digester (UltraClave MLS, Leutkirch, Germany) at 235°C for 60 min (1,000 W). If necessary, the samples were diluted with HNO_3_ 2% prior to analysis.

The sulfur content was determined with the Dumas method using an automated analyzer (Truman CNS, Leco, Germany). The samples were dried during 3 h at 105°C to determine the dry matter content and finally incinerated at 550°C until a stable mass was reached to determine the ash content in accordance with ISO 5984_2002 (prepASH, Precisa Gravimetrics AG, Dietikon, Switzerland).

The chloride content of the samples was determined argentometrically. The sample diluted with water was suspended with a Polytron mixer and titrated with silver nitrate after acidification with nitric acid. The end point of the titration was determined potentiometrically.

For the assessment of iodine content, a validated method for the extraction and analysis of iodine in liquids like milk was used (31). Briefly, iodine was measured after alkaline extraction with tetramethyl ammonium hydroxide (Trace Select, Honeywell Fluka, Switzerland) using sector-field inductively coupled plasma mass spectrometry (ICP-MS; Element XR, Thermo, Germany) with an iodine isotope dilution analysis with ^129^I (Standard Reference Material 4949C, National Institute of Standards and Technology, Gaithersburg, MD, USA). Initially, the ^127^I/^129^I-spike ratio was measured, and the updated value was applied in the subsequent calculations of the iodine concentration in the samples. The interference of ^129^Xe on the ^129^I signal was corrected according to the natural abundances of xenon.

The amounts of selenium and arsenic were measured in the samples after microwave-assisted pressure digestion in an autoclave (MLS Ultraclave III, Leutkirch, Germany) with nitric acid 60% (Ultrapur, Merck, Gernsheim, Germany). The reactor was pressurized, and the samples were digested at 220°C for 45 min. Subsequently, the mineralized solutions were transferred into polypropylene tubes (Sarstedt AG, Sevelen, Switzerland) and diluted to volume with pure water. An iCAP TQ ICP-MS (Thermo Scientific, Bremen, Germany) was used for the element analysis. Selenium and arsenic were acquired in a triple quadrupole mode, that is, a reaction cell pressurized with oxygen as a reactive gas; the first quadrupole set to selenium and arsenic masses; and the analysis quadrupole set to product ion masses (SeO^+^ and AsO^+^). The isotopes ^80^Se and ^75^As were used for actual calibration with ^103^Rh as internal standard (elemental standard solutions; Merck, Gernsheim, Germany).

### Assessments of sample contamination

The presence of two residues, glyphosate and aminomethylphosphonic acid (AMPA), in the samples were analyzed without derivatization using tandem mass spectrometry coupled to liquid chromatography as described by Zoller et al. (32). For the liquid samples tested, the limit of quantification (LOQ) and limit of detection (LOD) for both analytes were 0.3 and 0.1 ng mL^–1^, respectively.

### Contribution of various products to recommended dietary allowance

The contribution of the products to Recommended Daily Allowance (RDA) was calculated for adult women aged 19 to 65 years by using the reference values from Germany, Austria and Switzerland (DACH) (33). The adult women were chosen because it is a large age group with the same recommendations and this population group also represents the largest part of the vegetarian and vegan diet (5).

### Statistical analysis

Descriptive statistics using mean and standard deviation (for products with *n* > 1) were calculated using R^1^ from at least two independent measurements. Figures were created with the R-library ggplot2.

Statistical analyses were performed using analysis of variance (ANOVA) for selected ingredients (vitamins and minerals), and the level of significance was set at *P* < 0.05. The different products were treated as factors. Hemp and spelt were sometimes grouped together in a group called “other.” Further ANOVA were performed for either the dependant variables calcium and iodine (see chapter “Minerals and trace elements”) or vitamins (pantothenic acid, vitamin B_2_, vitamin B_12_, and vitamin D_2_, see chapter “Vitamins”), using “milk” as the main focus and by grouping the alternative milk products either in groups with or without fortification of calcium-phosphate or Lithothamnium algae or in groups with or without supplementation of vitamins or sunflower oil, according to the general information on the packaging. Another series of ANOVA were performed by regrouping the products into groups of “milk,” “soy,” and “other” for the vitamins (vitamin E and folic acid, see chapter “Vitamins”).

When the ANOVA test result was significant, Tukey multiple pairwise comparisons were performed while adjusting the *P* value for multiple testing.

## Results

### Characteristics of the plant-based drinks

A total of 27 plant-based drinks and two bovine whole milk (3.5% fat) samples were included in the analysis. Whole milk products (*n* = 2) were used to compare the fat quality of the beverages as well. The varieties of the plant-based beverages analyzed were almond (*n* = 4), cashew (*n* = 2), coconut (*n* = 3), hemp (*n* = 1), oat (*n* = 4), rice (*n* = 5), soy (*n* = 7), and spelt (*n* = 1). The compositions according to the labels of the different samples are listed in Supplementary Table 1.

To all but four samples (three soy drinks and one rice drink), salt (sea, table, and cooking salts) was added. Nine samples contained added sugar or sweeteners, and 14 samples contained stabilizers, emulsifiers, and thickeners. All rice and oat samples and the hemp and spelt samples contained sunflower oil. Nine samples were supplemented with one or more vitamins and calcium. In four samples, calcium was added in the form of red algae (*Lithothamnium calcareum*; Supplementary Figures 2, 3). Two of the coconut drinks were blended with small amounts of rice flour, and two of the rice drinks were fermented.

The dry matter content in the individual plant-based drink samples ranged from 25.24 g kg^–1^ (almond drink) to 133.67 g kg^–1^ (rice drink; Table 1). The highest mean values of dry matter in the plant-based drinks were found in the rice drinks, followed by the oat, soy, cashew, and coconut drinks. As only one sample was analyzed for hemp and spelt, no mean values could be calculated for these two varieties, “leaving them in the middle position”. In comparison with all the plant-based drinks, milk has a mean dry matter value of 121.6 ± 1.2 g kg^–1^, the highest amongst the values of all the varieties analyzed. Because of high variability of the dry matter in the different products, the comparison of the compositions of the individual drinks with milk was based on volume.

**TABLE 1:** Mean values and ranges or single values of energy, dry matter, total protein, sum of amino acids, total fat, and total carbohydrate contents in the different plant-based drinks and cow’s milk.

Product	n	Energy^1)^ Kcal kg^–1^		Energy^2)^ Kcal kg^–1^		Dry matter g kg^–1^		Total protein g kg^–1^		Sum of AAs g kg^–1^		Total fat g kg^–1^		Total carbohydrates g kg^–1^	
		Mean	Min/Max	Mean	Min/Max	Mean	Min/Max	Mean	Min/Max	Mean	Min/Max	Mean	Min/Max	Mean	Min/Max
Almond drink	4	328	130/600	324	129/616	54.6	25.2/99.0	10.2	5.4/18.3	8.1	4.6/15.3	25.6	11.0/49.6	13.0	1.9/24.2
Cashew drink	2	415	340/490	393	322/464	69.0	51.8/86.1	13.3	12.1/14.5	11.5	10.0/13.1	27.6	26.0/29.3	22.8	9.9/35.6
Coconut drink	3	230	120/370	215	139/325	43.0	28.8/57.0	3.2	1.2/6.1	2.5	0.9/5.0	10.2	1.3/19.7	27.7	22.7/34.6
Cow’s milk	2	655	640/670	649	644/654	121.6	120.8/122.4	32.6	32.6/32.7	33.0	32.9/33.1	35.4	34.9/35.9	50.3	49.9/50.7
Hemp drink	1	310	-	386	-	59.6	-	7.2	-	5.4	-	32.6	-	16.0	-
Oat drink	4	415	390/450	323	168/441	90.3	86.3/98.0	4.6	2.8/5.8	4.0	2.2/5.4	14.5	13.4/15.4	36.7	6.1/69.7
Rice drink	5	546	480/680	313	224/396	118.4	111.9/133.7	1.7	0.7/3.0	1.3	0.4/2.5	12.6	9.8/21.8	48.2	27.7/73.2
Soy drink	7	416	350/520	396	328/462	84.2	63.1/102.6	37.8	34.0/48.0	34.6	29.4/43.2	20.6	16.1/28.2	14.7	2.6/33.8
Spelt drink	1	420	-	297	-	86.9	-	7.2	-	5.9	-	12.2	-	49.5	-

^1)^Energy according to label; ^2)^Energy calculated from content of fat, protein, and carbohydrates.

The highest average energy content according to the label was reported for the rice drinks, followed by the soy, spelt, and cashew and oat drinks, respectively. The almond, hemp, and coconut drinks contained the lowest amounts of calories. The mean energy values of the plant-based drinks were all lower than that of milk (655 kcal kg^–1^; Table 1). A comparison with the calculated values using the concentrations of protein, fat and carbohydrates shows good correlation with most of the drinks except rice, spelt, hemp and oat (Table 1). It is possible that other carbohydrates are present in these products than those we analyzed.

### Macronutrients in plant-based drinks and cow’s milk

#### Protein

The crude protein contents of the plant-based drinks ranged from a minimum of 0.6 g kg^–1^ in a rice drink to 43.0 g kg^–1^ in a soy drink. The highest mean protein contents in the plant-based drinks were found in the soy drinks, followed by the cashew and almond drinks (Table 1). The mean protein values in the oat-, coconut-, and rice-based beverages were very low. The values in the individual samples of spelt and hemp drinks were both at an in-between level (Table 1). The mean crude protein content of milk was 32.6 g kg^–1^ and thus was within the range for soy-based beverages. Total amino acids included the 9 essential amino acids (eAA; histidine, isoleucine, leucine, lysine, methionine, phenylalanine, threonine, tryptophan, and valine), 2 semi-essential amino acids (cysteine and tyrosine), and seven non-essential amino acids (alanine, arginine, aspartic acid, glutamic acid, glycine, proline, and serine). The individual contents of the amino acids were highly variable between the plant-based drinks and milk, and amongst the individual varieties (Supplementary Table 3).

Small amounts of γ-aminobutyric acid were detected in 13 plant-based samples, of which only a significant value of 0.29 g L^–1^ was measured in a coconut drink. In all other drinks, the concentrations were very low (0.01–0.03 g L^–1^) or zero as in milk. Taurin was not detected in any of the products.

**FIGURE 1:**
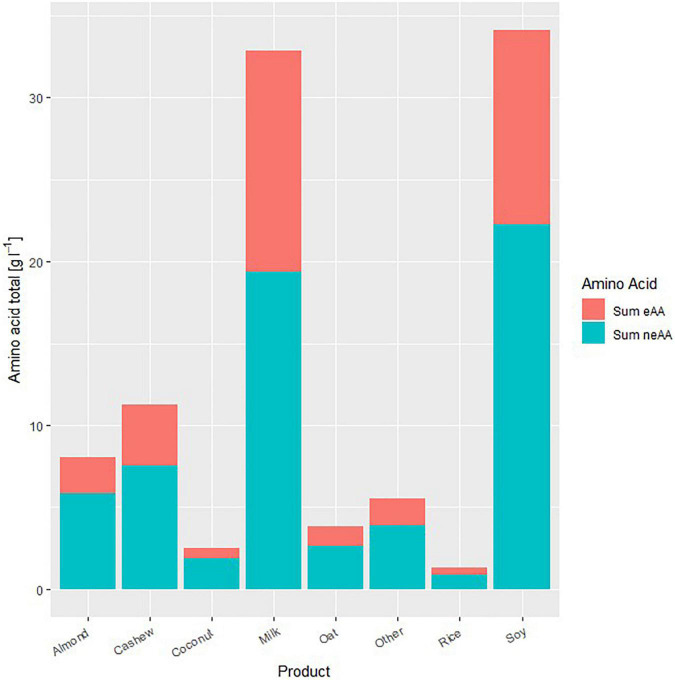
Mean amino acid composition of the sampled plant-based beverages and milk (g L^–1^). eAA, essential amino acids (blue); neAA, non-essential amino acids (red).

As for the total protein content, the soy drinks were closest to milk in their free amino acid concentrations, with essential amino acids having the highest mean value (35.1% ± 0.2%). In all other plant-based varieties, the concentrations of the individual amino acids were lower than those in the soy drinks or milk owing to the lower protein content and poorer ratio of essential amino acids to non-essential amino acids, resulting in a lower biological value of their proteins (Figure 1).

The sums of the individual amino acids are presented in Supplementary Table 3. The values correspond well for cashew, oat, soy, and spelt. For almond, coconut, hemp and rice the differences to the measured crude protein range between 10 and 20%. This is due to the conversion factor of 5.6 applied to all plant-based products. As expected, this overestimates the protein content of these plant-based drinks. For milk, the sum of amino acids agrees with the measured values of crude protein (Table 1).

The calculation of the DIAAS for the evaluation of protein quality showed the high quality of milk protein and the lower quality of all plant-based proteins (Supplementary Table 4). The DIAAS of milk for children aged > 3 years, adolescents, and adults are > 100% for all essential amino acids. Of the plant-based drinks evaluated, only the soy drinks had a DIAAS of > 100% for these age groups. All the other drinks had DIAASs between 39.0% (almond) and 78.8% (cashew). The DIAAS of milk for children aged 6 months to 3 years showed a value > 100%, with the first limiting indispensable amino acids (IAAs) for cysteine and methionine (sulfur amino acids). All plant-based proteins were inadequate in 2 (soy) to 8 (almond, rice, coconut, and hemp) IAAs and therefore of much lower quality than milk for young children. For this age group, soy drinks had the highest DIAASs amongst all plant-based drinks (91.9%) and had the same limiting IAAs as milk (Supplementary Table 4).

For infants (up to 6 months old) DIAAS for milk is 65.4 with tryptohane as the first limiting amino acid. For this youngest age group, all plant-based proteins had inadequate DIAAS values ranging from 27.1% (almond) to 71.2% (soy; Supplementary Table 4).

#### Fat and fatty acids

The highest mean values of fat in plant-based drinks were found in cashew drinks (27.6 ± 2.3 g kg^–1^), closely followed by almond and soy drinks. The oat, rice, and coconut drinks and the single sample of spelt drink were distinctly lower in fat, whereas the hemp drink (32.6 g kg^–1^) had a high fat concentration, similar to the mean value in milk (35.4 ± 0.7 g kg^–1^; Table 1).

The fatty acids found in the plant-based drinks belong almost exclusively to long-chain fatty acids, except those in coconut fat. Only the coconut drinks and milk contain short-chain fatty acid and relatively high amounts of medium-chain fatty acid. The coconut drinks contained the highest amount of SFA, followed by milk. In all other plant-based drinks, the FAs belonged to the long-chain group and were mainly monounsaturated and polyunsaturated (Supplementary Table 5). High levels of omega-6 fatty acids were found in these drinks compared with coconut drink or milk. Conversely, omega-3 was detected only in substantial amounts in soy drinks and milk. Consequently, the mean ratio of omega-6 to omega-3 FAs was relatively low in the milk and soy drinks compared with the other plant-based drinks (Table 2).

**TABLE 2:** Calculated values for the ratio of omega-6 to omega-3 fatty acids in different plant-based drinks and cow’s milk.

Product	Ratio omega-6 to omega-3
Almond drink (*n* = 4)	(127 to 235): 1
Cashew drink (*n* = 2)	78:1
Coconut drink (*n* = 3)	(11 to 18): 1
Cow’s milk (*n* = 2)	(2 to 3):1
Hemp drink* (*n* = 1)	30: 1
Oat drink* (*n* = 4)	(84 to 105): 1
Rice drink* (*n* = 5)	(92 to 175): 1
Soy drink (*n* = 7)	(7 to 8): 1
Spelt drink* (*n* = 1)	112: 1

Ranges are given in brackets, *Beverages to which sunflower oil was added according to the label.

The mean total trans fatty acid contents ranged from 0.0015 g 100 g^–1^ (coconut) to 0.022 g 100 g^–1^ (hemp) for the vegetable drinks and 0.14 g 100 g^–1^ for milk. The mean total conjugated amino acid (CLA) content of the plant-based drinks ranged from 0.0003 g 100 g^–1^ (coconut drink) to 0.004 g 100 g^–1^ (hemp drink), while milk provided a mean CLA content of 0.035 g 100 g^–^1 product (Supplementary Table 5).

#### Carbohydrates

In all plant-based drinks and milk, the following carbohydrates were measured: sucrose, fructose, glucose, lactose, starch, and fiber. As fiber concentration was generally very low in all samples, these results are not reported herein.

The total carbohydrate contents of the plant-based drinks ranged from 1.9 g kg^–1^ in an almond drink to 73.2 g kg^–1^ in a rice drink. The total carbohydrate content was high in the spelt, rice, and oat drinks; moderate in the coconut and cashew drinks; and low in the soy, almond, and hemp drinks (Table 1). The highest total carbohydrate concentration was detected in milk (50.2 ± 0.53 g kg^–1^).

Unlike milk, whose sole source of carbohydrate is lactose, plant-based drinks contain sucrose and glucose as the main types of carbohydrates. Most plant-based drinks with high sucrose concentrations had low glucose contents and vice versa, and all plant-based drinks and milk contained very low fructose concentrations (Table 3).

**TABLE 3:** Mean values and ranges of sucrose, fructose, glucose, lactose, and starch contents in the different plant-based drinks and cow’s milk.

Product	n	Sucrose	Min/Max	Fructose	Min/Max	Glucose	Min/Max	Lactose	Min/Max	Starch	Min/Max
		g kg^–1^	g kg^–1^	g kg^–1^	g kg^–1^	g kg^–1^	g kg^–1^	g kg^–1^	g kg^–1^	g kg^–1^	g kg^–1^
Almond drink	4	12.5	1.6/23.4	0.01	0.0/0.04	0.1	0.0/0.1	-	-	0.4	0.0/0.8
Cashew drink	2	3.6	3.5/3.7	0.0	0.0	0.3	0.1/0.4	-	-	19.0	6.1/31.8
Coconut drink	3	16.0	0.9/33.5	0.0	0.0	3.1	0.1/9.0	-	-	8.6	0.9/12.8
Cow’s milk	2	0.0	0.0	0.0	0.0	0.1	0.1	50.2	49.9/50.6	0.0	0.0
Hemp drink	1	1.35	-	0.0	-	0.3	-	-	-	14.4	-
Oat drink	4	1.5	0.8/3.0	0.9	0.0/2.1	33.2	1.5/58.1	-	-	7.9	1.7/10.7
Rice drink	5	2.3	0.2/10.1	0.8	0.1/1.8	24.6	6.7/42.5	-	-	20.5	8.4/31.2
Soy drink	7	14.0	2.2/32.6	0.01	0.0/0.1	0.1	0.0/0.1	-	-	0.7	0.4/1.2
Spelt drink	1	0.7	-	1.1	-	37.2	-	-	-	1.3	-

The starch content ranged from 0.0 g kg^–1^ in an almond drink to 31.8 g kg^–1^ in a cashew drink (Table 3). The highest mean total starch content in the plant-based drinks was found in the rice drinks, followed by the cashew, hemp, coconut, and oat drinks. In the spelt, soy, and almond drinks, only small mean concentrations of starch were detected. As expected, no starch was present in milk (0.0 g kg^–1^).

#### Micronutrients in plant-based drinks and milk

##### Vitamins

Of the plant-based drinks, seven were fortified with different vitamins according to label (Supplementary Table 1 and Supplementary Figure 1). Even when fortification was taken into account, the cashew, coconut, oat, and rice drinks generally contained low amounts of vitamins compared with the other plant-based drinks, and vitamins C, A, and K_2_ could only be detected in milk (Table 4). However, provitamin A content, which was to be expected in the plant-based drinks, was not analyzed.

**TABLE 4:** Mean values and range of vitamins analyzed for different plant based drinks and cow’s milk.

Vitamin	Unit		Almond drink		Cashew drink		Coconut drink		Cow’s milk		Hemp drink	Oat drink		Rice drink		Soy drink		Spelt drink
		N	4		2		3		2		1	4		5		7		1
			Mean	Min/Max	Mean	Min/Max	Mean	Min/Max	Mean	Min/Max	Mean	Mean	Min/Max	Mean	Min/Max	Mean	Min/Max	Mean
C	μg 100 g^–1^		n.d.	-	n.d.	-	n.d.	-	202.3	0.0/404.6	n.d.	n.d.	-	n.d.	–	n.d.	-	n.d.
Biotin	μg 100 g^–1^		0.6	0.4/0.8	0.7	0.4/0.9	0.7	0.2/1.7	1.7	1.5/2.0	1.4	1.3	1.1/1.6	0.3	0.2/0.4	1.4	0.8/2.3	0.6
Niacin	μg 100 g^–1^		159.3	85.6/267.1	82.5	67.3/97.6	100.1	24.3/208.5	132.4	124.9/139.9	219.4	60.7	49.1/81.5	132.4	115.5/153.8	174.2	90.2/283.8	230.2
Pantothenic acid	μg 100 g^–1^		17.9	5.8/31.5	94.1	81.8/106.4	29.7	14.8/49.3	357.9	329.8/386.1	152.6	145.4	134.9/157.2	130.4	96.2/187.9	118.3	73.8/192.0	90.0
B1	μg 100 g^–1^		6.3	3.8/10.1	17.7	12.1/23.2	3.3	1.9/4.7	11.9	11.7/12.0	16.3	25.2	19.4/35.7	5.2	3.1/10.2	43.5	21.8/86.6	21.9
B2	μg 100 g^–1^		54.9^1)^	14.9/136.8	8.8	8.1/9.6	0.5	0.1/1.4	108.3	107.9/108.7	20.4	14.0^1)^	5.9/34.9	1.3	0.8/1.6	57.8^1)^	8.4/218.6	6.3
B6	μg 100 g^–1^		2.7	1.1/4.0	9.2	6.2/12.1	3.9	1.4/8.6	20.1	18.1/22.1	19.2	5.0	4.0/6.6	4.3	2.8/5.4	20.6	12.8/29.6	19.1
B12	μg 100 g^–1^		0.2^1)^	0.0/0.6	n.d.	-	0.03^1)^	0.0/0.1	0.2	0.2	n.d.	0.1^1)^	0.0/0.3	n.d.	-	0.1^1)^	0.0/0.3	0.1
Folic acid	μg 100 g^–1^		1.9	0.8/3.9	3.4	3.0/3.8	0.5	0.4/0.7	3.2	1.2/5.2	0.2	2.3	2.0/2.5	1.6	1.2/2.0	17.7	10.8/23.6	0.1
A	μg 100 g^–1^		n.d.	-	n.d.	-	n.d.	-	29.2	27.5/30.8	n.d.	n.d.	-	n.d.	-	n.d.	-	n.d.
E	μg 100 g^–1^		1101.6^1)^	419.3/2304.5	304.0	210.9/397.2	n.d.	-	89.1	85.1/93.1	1751.2^2)^	513.7^2)^	380.2/693.2	457.7^2)^	382.6/558.2	2822.0	1759.8/3865.8	443.8^2)^
D2	μg 100 g^–1^		0.4^1)^	0.0/1.2	n.d.	-	0.2^1)^	0.0/0.5	n.d.	-	0.2	0.3^1)^	0.0/1.1	n.d.	-	0.4^1)^	0.0/1.0	0.1
K1	μg 100 g^–1^		n.d.	-	1.8	1.0/2.6	n.d.	-	0.2	0.1/0.2	0.2	0.1	0.1	0.03	0.0/0.1	3.5	2.1/4.8	0.1
K2	μg 100 g^–1^		n.d.	-	n.d.	-	n.d.	-	0.4	0.4	n.d.	n.d.	-	n.d.	-	n.d.	-	n.d.

n.d., not detected; ^1)^contains products supplemented with vitamins; ^2)^contains products supplemented with sunflower oil.

Among the plant-based drinks, the hemp-based beverage was highest in biotin and pantothenic acid contents. The soy drinks were highest in vitamin B_1_, B_2_, and B_6_; folic acid; and vitamins E and K_1_ contents. The almond drinks had the highest vitamin B_12_ and D_2_ contents (with both vitamins being fortified), and the spelt drink had the highest niacin content.

All measured vitamins were detected in milk, except vitamin D_2_. The milk samples showed higher contents of naturally occurring (e.g., with no fortification) vitamins C, A, K_2_, B_2_, biotin, pantothenic acid, and vitamin B_12_ than the plant-based beverages. Several plant-based drinks were fortified with vitamins such as vitamins B_2_, B_12_, E, and D_2_ (Table 4). However, milk had significantly higher pantothenic acid content than all plant-based drinks (*p* < 0.05) and significantly higher vitamins B_2_ and B_12_ than the unfortified plant-based drinks (*p* < 0.05). As the variation amongst the drinks fortified with vitamins B_2_ and B_12_ was relatively high, no significant differences were found between these products and milk. Owing to the fortification of vitamin D in some drinks, they became significantly richer in this vitamin than milk and the non-fortified drinks (*p* < 0.5). Furthermore, in some of the plant-based drinks such as hemp (1), oat (4), rice (5), and spelt (1), sunflower oil was added and therefore likely contributed to increasing the vitamin E level (51.75 mg 100 g^–1^) (34), as sunflower oil is naturally rich in this vitamin (Supplementary Table 1). Thus, these drinks had higher vitamin E content than milk but lower vitamin E content than soy drinks. The soy drinks also provided significantly higher amounts of folic acid than milk, and all other plant-based drinks (*p* < 0.05).

Except for vitamin C and folic acid, the variability of the vitamin contents in the two measured milk samples appeared relatively low. In the plant-based drinks, however, the contents of the individual vitamins may greatly vary. For example, the variabilities of niacin (almond, coconut, and soy), pantothenic acid (rice and soy), vitamin B_1_ (soy), vitamin B_2_ (almond, oat, and soy), and vitamin E (almond, cashew, oat, and soy) within the same variety were noticeably high (Table 4 and Supplementary Figure 1).

##### Minerals and trace elements

The rice, oat, spelt, and coconut drinks were generally low in minerals, whereas the soy drinks provided significant amounts of all minerals except sodium, iodine, and chloride (Table 5). The cashew drinks were high in copper (1.29 ± 0.43 mg kg^–1^), zinc (3.04 ± 1.76 mg kg^–1^), and selenium (21.01 ± 14.0 μg kg^–1^); the hemp drink provided considerable amounts of manganese (1.22 mg kg^–1^), copper (0.81 mg kg^–1^), and selenium (13.42 μg kg^–1^) and similar sodium level (567 mg kg^–1^) as the almond drinks (524 mg kg^–1^).

**TABLE 5:** Mean values and range of minerals and trace elements analyzed for different plant-based drinks and cow’s milk.

Mineral	Unit		Almond drink		Cashew drink		Coconut drink		Cow’s milk		Hemp drink	Oat drink		Rice Drink		Soy drink		Spelt drink
		N	4		2		3		2		1	4		5		7		1
			Mean	Min/Max	Mean	Min/Max	Mean	Min/Max	Mean	Min/Max	Value	Mean	Min/Max	Mean	Min/Max	Mean	Min/Max	Value
P	mg kg^–1^		434	110/660	337	230/450	296	50/660	924	870/980	266	289	110/730	71	30/130	807	460/1300	310
Na	mg kg^–1^		524	250/850	306	190/430	335	260/440	381	370/390	567	395	310/460	149	30/320	229	10/430	440
Mn	mg kg^–1^		0.39	0.22/0.80	1.05	0.43/1.66	0.34	0.30/0.39	n.d.	-	1.22	0.15	0.0/0.32	0.08	0.0/0.41	2.16	1.36/2.86	0.47
Mg	mg kg^–1^		95	60/170	158	110/210	59	30/90	100	100	76	42	20/70	68	30/100	200	130/270	72
K	mg kg^–1^		342	170/630	454	440/470	723	140/1800	1615	1580/1650	402	296	270/340	307	100/590	1643	940/2930	419
Fe	mg kg^–1^		1.21	0.72/2.22	2.95	1.86/4.04	0.62	0.31/0.86	n.d.	-	2.08	0.83	0.0/1.94	1.42	0.0/2.42	5.93	3.29/9.86	0.66
Cu	mg kg^–1^		0.47	0.21/0.93	1.29	0.98/1.59	0.26	0.0/0.57	n.d.	-	0.81	0.07	0.0/0.27	n.d.	-	1.33	1.02/1.72	0.27
Ca	mg kg^–1^		656^1)^	50/1250	64	60/70	471^1)^	30/1330	1121	1090/1150	45	499^1)2)^	20/1330	544^2)^	50/1040	842^1)2)^	80/1670	121
Zn	mg kg^–1^		1.33	0.62/2.74	3.04	1.8/4.28	0.36	0.24/4.23	3.42	3.37/3.48	1.49	0.28	0.0/0.53	0.53	0.4/0.73	3.40	2.4/4.43	0.80
Se	μg kg^–1^		1.58	0.78/2.67	21.01	11.12/30.91	3.69	0.48/7.53	16.21	13.5/18.9	13.42	1.60	1.18/2.42	0.86	0.52/1.12	10.48	3.12/22.69	2.88
S	mg kg^–1^		68	40/100	138	110/160	75	30/150	305	300/310	92	79	50/90	38	30/50	301	240/350	87
I	μg kg^–1^		3.75	2.5/5.2	4.95	2.6/7.3	6.20	2.8/9.0	115.70	81.8/149.6	3.4	12.83^2)^	0.2/43.9	21.16^2)^	0.2/39.4	15.13^2)^	0.2/76.6	3.9
Cl	mg kg^–1^		686	410/900	348	60/640	678	470/860	980	980	750	639	550/700	579	140/960	119	0/430	690

n.d., not detected; ^1)^contains with calcium phosphate supplemented products; ^2)^contains with Lithothamnium calcareum supplemented products.

Compared with milk, the plant-based drinks contained lower amounts of phosphorus, potassium, calcium, zinc, sulfur, iodine, and chloride. No iron, copper, and manganese were detected in milk (Table 5). Some drinks made of almond, coconut, oat, rice, and soy were fortified with tricalcium phosphate or calcium containing algae (*L. calcareum*; Supplementary Table 1). All drinks without fortification had significantly lower calcium contents than milk (*p* < 0.05).

The addition of seaweed (*L. calcareum*) improved the calcium concentration to a level comparable with that in milk but significantly lower than those in drinks supplemented with tricalcium phosphate (*p* < 0.05). The addition of seaweed also seemed to elevate the iodine content. This was the case in a soy drink, an oat drink, and three rice drinks (Supplementary Table 1). However, the concentrations remained significantly below those in milk (*p* < 0.05). The variabilities of the iron, zinc, calcium, potassium, and selenium contents within the same variety were quite high (Table 5 and Supplementary Figure 2).

##### Toxic residues

The arsenic concentration was low in all plant-based drinks and milk (ranging from 2.0 to 4.6 μg kg^–1^), except for rice drinks, in which elevated concentrations ranging from 10.2 to 12.4 μg kg^–1^ were measured (Supplementary Table 6).

Only traces or concentrations below the LOQ of glyphosate and AMPA were detectable in most samples (Supplementary Table 6). None of the milk samples contained glyphosate or AMPA levels higher than the LOD. In 3 soy, 2 almond, 1 rice, and 1 oat drink and in the hemp drink, glyphosate or AMPA concentrations higher than the LOQ (0.3 μg L^–1^) were detected, but the levels were relatively low (between 0.3 and 0.8 μg L^–1^).

#### Contribution to dietary recommendations

One portion (200 mL) of milk contributes to an average of > 10% of the RDA for biotin (11.5%, 9.8–13.3%); pantothenic acid (11.9%, 11.0–12.9%); vitamin B_2_ (19.7%, 19.6–19.8%); minerals and trace elements, including phosphorus (26.4%, 24.9–27.9%), calcium (22.4%, 21.9–23.0%), and iodine (15.4%, 10.9–20.0%); and macronutrients, namely protein (13.6%, 13.6%) and fat (11.3%, 11.2–11.5%; Supplementary Table 7). The variability of the contents of the milk samples was low, except for iodine.

One portion (200 mL) of soy drink contributes an average of > 10% of the RDA for vitamin B_2_ (10.5%, 1.5–39.7%); folic acid (23.5%, 14.4–31.4%); vitamin E (47.0%, 29.3–64.4%); vitamin D_2_ (11.4%, 0–13.7%); vitamin K_1_ (11.5%, 7.1–15.9%); minerals and trace elements, including phosphorus (23.1%, 13.1–37.0%), manganese (12.3%, 7.8–16.4%), magnesium (13.4%, 8.7–18.2%), copper (21.3%, 16.3–27.4%), and calcium (16.8%, 1.7–33.5%); and the macronutrient protein (14.1%, 11.7–17.9%; Supplementary Table 7).

All other plant-based drinks had < 10% contributions of most nutrients, except the almond, oat, and rice drinks for calcium (13.1%, 1.1–25.0%; 10.0%, 0.3–26.7%; and 10.9%, 1.0–20.8%, respectively), almond and hemp for vitamin E (18.4%, 7.0–38.4% and 29.2%, respectively), almond for phosphorus (12.4%, 3.1–18.7%), cashew for magnesium and copper (10.6%, 7.4–13.7% and 20.8%, 15.7–25.5%), and hemp for vitamin K_1_ (10.6%), copper (13.0%), and fat (10.4%; Supplementary Table 7). However, the individual plant-based beverages differed considerably in their respective contributions.

## Discussion

Plant-based drinks are considered alternatives to milk and are often displayed close to milk products in the stores. They are often touted as better tolerated, healthier, and more sustainable than milk. To compare the nutritive value of these plant-based drinks to milk, it is important to know the quantitative composition of macro- and micronutrients of these products as accurately as possible. The results of this study provide a snapshot of quantitative information about macro/micronutrient profiles and residues from products commercially available in Switzerland at the time of this investigation. They can be used to increase awareness about the possible nutritional gaps when proceeding with total milk dietary exclusion and to optimize dietary plans able to adequately fulfill nutritional requirements. This is important for the nutritional guidance of the general population but particularly for people affected by specific clinical conditions (e.g., allergy or intolerance to cow’s milk proteins, lactose intolerance, galactosemia and post-infection diarrhea) and during complementary feeding (35). In the following, we discuss nutrient composition of the measured plant-based drinks and cow’s milk highlighting their peculiarities as regards with nutritional requirements. Foremost, we observed that plant-based drinks exhibited lower dry masses indicating lower macronutrient and micronutrient density making the ratio of the cost per amount of nutrients less advantageous in plant-based drinks than in cow’s milk (17).

### Macronutrient profile

Cow’s milk allergy (CMA) prevalence is estimated to be at 2–3% and < 1% amongst children and adults, respectively (36–39). CMA manifests itself in various symptoms and is mainly induced by casein and β-lactoglobulin making plant-based drinks interesting alternatives. However, Jeske et al. (40) reported that 14% of CMA people also experience soy protein allergy and should then avoid soy drinks (40). Immune reactivity to almond and coconut drinks was also reported (8). In CMA affected infants, tolerance to soy proteins needs to be established and substitution of cow’s milk-based formula is only recommended based on age (41). Most plant-based drinks provide less calories but also less protein contents than milk (Table 1). Exceptions are soy-based drinks, whose protein contents are equal or even slightly higher (31.2–48 g kg^–1^) than those of milk (32.6 g kg^–1^). Even though protein intake is on average good in Western countries, some population groups such as infants, children and elderly have higher protein and amino acid needs and it is important to ensure bioavailability of amino acids with good protein quality (42, 43). Indeed, quality of plant proteins is often reduced relatively to animal proteins due to poorer digestibility, occurrence of anti-nutritional factors, lower essential amino acid content (especially leucine), and deficiency in other essential amino acids such as sulfur amino acids or lysine (44, 45). The Digestible Indispensable Amino Acid Score (DIAAS) method, based on the ileal digestibility of individual indispensable amino acids in relation to the amino acid reference pattern for human requirements, is nowadays seen as a standard for protein quality determination as recommended by FAO (21, 46, 47). The DIAAS also considers the effect of anti-nutritive compounds that interfere with digestion, such as phytic acid, polyphenols, and protease inhibitors. Even if clinical evidence linking DIAAS with clinical health outcomes remains in development, consumption of proteins with low DIAASs was associated with lower muscle protein synthesis (44). Our data show superior protein quality as measured with DIAAS as well as higher contents of essential amino acids (including lysine and methionine) and essential to total amino acid ratio of milk relatively to plant-based drinks except for soy drinks for the 0 to 6 month age group (Supplementary Tables 3, 4). Literature on DIAAS from plant-based beverages is scarce and limited to original protein sources as opposed to final processed products (12). Thus, DIAASs from cow’s milk protein (1.18/1.16), soy (0.90/0.91/0.89), rice (0.59/0.49), oat (0.54/0.57), hemp (0.54), and almond proteins (0.40) were published (48–50). Whereas our DIAASs data for milk (1.24), soy drinks (0.92), and oat drinks (0.50) are congruent with literature for the same age group (0.5-to 3.0-year-old children), values for rice (0.36), almond (0.33), and hemp drinks (0.47) appeared lower than the ones previously reported (48–50). No comparative DIAAS values for coconuts and spelt proteins could be found and to our knowledge only a value < 50% was indicated for coconut drinks (16).

Not surprisingly, fatty acid profiles from plant-based drinks show higher contents of mono-and poly-unsaturated fatty acids relatively to milk, except for coconut beverages. Milk is particularly rich in saturated fatty acids and concerns were raised about how its high consumption could associate with cardiometabolic health and diabetes (51–53). Nowadays, national and international recommendations to regulate total and saturated fat consumptions are available (54, 55) and causality between total fat, saturated fatty acids, or dairy products and cardiometabolic health remains unestablished (52, 53, 56). Milk also contains 0.31–0.38 g of conjugated linoleic acid per 100 g fat that is reported with positive effects on health (57). The omega-3 fatty acids, especially those originating from seafood and plants are reported with beneficial and anti-inflammatory effects (52, 58). In particular, alpha linolenic acid (essential omega-3 fatty acid) owns capacity for enzymatic conversion to beneficial eicosapentaenoic and docosahexaenoic acids. As the pro-inflammatory omega-6 fatty acids compete for the same enzyme system as omega-3 fatty acids, the lowest possible ratio of omega-6 to omega-3 is reported advantageous (58). According to our calculation, the lowest ratio was obtained for milk at (2 to 3):1, followed by the soy (7 to 8):1 and coconut drinks (11 to 18):1 (Table 2). All other drinks provided much higher ratios ranging from 30:1 (hemp) to 127 to 235:1 (almond) (Table 2). One can hypothesize that the addition of sunflower oil to plant-based drinks contributes to the increase of omega-6 to omega-3 ratio (59).

Lastly about macronutrients, key advantage of plant-based drinks relies with the absence of lactose making them suitable when in presence of lactose intolerance (12). Nevertheless, processing solution exists to eliminate lactose from cow’s milk. In Western diets, excessive consumption of simple sugars (glucose, sucrose) with high glycaemic index (GI) link with increased risks of obesity, cardiovascular disease, and type 2 diabetes over time (60). Schwingshackl and Hoffmann recommended to limit the intake of high GI foods (with GI > 70) not only in diabetic or prediabetic individuals but also for prevention (61). Jeske et al. determined the glycaemic index (GI) of 17 plant-based drinks and milk (40). Whereas milk was assigned a GI of 46.93, plant-based alternatives exhibited variable GI ranging from 47.53 to 99.96. Our data show that sucrose was the main sugar in the plant-based drinks with a GI of 61 exceeding then the milk lactose GI of 46 (62). The variability of sucrose level in plant-based drinks was also noticeable with 0.2 g kg^–1^ (rice drink) up to 33.5 g kg^–1^ (coconut drink). Furthermore, several plant-based drinks have high starch contents (rice and cashew drinks), which contributes to increasing GI due to hydrolysis into glucose by digestive enzymes (63). Despite the generally lower total carbohydrate content in plant-based drinks (except some rice and oat drinks), milk consumption appears therefore more favorable in terms of GI. Regarding the fibers that were not reported in this study, oat-based drink is likely to have the highest contents with reported up to 0.5 g/100 mL (64). By contrast, milk contains only trace amounts of oligosaccharides, which might provide a prebiotic function (65). Two rice drinks were described as fermented, which might have changed carbohydrate composition and possibly increased glucose levels. It would be interesting to check whether these products were processed with enzymatic treatment or fermented with bacteria.

### Micronutrient profile

While milk provides the full range of vitamins, vitamins C, A, and K_2_ could not be detected in the measured plant-based drinks. It is worth mentioning that carotenoids (provitamin A), naturally occurring in plants, were not included in our analyses and therefore our results might underestimate contribution of plant-based drinks to vitamin A status (66). Several vitamins including B_2_, B_12_, E, and D_2_ were added in plant-based drinks with B_2_, B_12_, and D_2_ almost absent in non-fortified products (Table 4 and Supplementary Table 1). As expected, the vitamin E contents of most plant-based beverages were higher than those of cow’s milk, with the soy and almond drinks providing the highest contributions to the RDA for this vitamin reaching up to 47% of RDA (Supplementary Table 7). Both almond and soy are naturally high in vitamin E (34, 67). The vitamin E concentrations in the rice, oat, and spelt drinks might have benefited from the addition of sunflower oil that is known to be naturally rich in vitamin E (34). Furthermore, the soy and cashew drinks provided significant concentrations of vitamin K_1_ (phylloquinone) but vitamin K_2_ (menaquinone) could only be measured in cow’s milk, which was in agreement with earlier results (68). It was reported that intake of menaquinone, even in small amounts, can significantly contribute to vitamin K status thanks to higher bioavailability versus phylloquinone (69). It is important to note that food processing conditions for the production of plant-based drinks may reduce vitamin concentrations and particularly the levels of the heat-sensitive vitamins C, B_1_, and A (70–72).

The analysis of minerals and trace elements also reveals several peculiarities of plant-based drinks versus cow’s milk. Milk is a well-known natural source of calcium and 13 out of 27 plant-based beverages were fortified in this element either using (tri)calcium phosphate or *L. calcareum* (red algae) (Supplementary Table 1). However, the bioavailability of tricalcium phosphate was shown to be 25% less in a soy-based beverage relatively to milk calcium (73). Furthermore, the use of red algae that shows similar properties as calcium carbonate raises discussions about its ecological impact (74). The calcium-to-phosphorous ratio for the non-fortified beverages were quite low compared with cow’s milk and high calcium-to-phosphate ratios are better for bone health (75, 76). Plant-based drinks naturally contain phytic acid as main source of phosphorous, and require thus calcium fortification (for example tricalcium phosphate) to increase the ratio (77). However, phytic acid is a known antinutrient able to chelate micronutrients such as calcium, zinc, magnesium and iron preventing mucosal absorption and therefore limiting the bioavailability of these minerals. Within plant-based drinks, soy drinks exhibited highest levels of several minerals delivering on average 21.3% (copper), 13.4% (magnesium), 12.3% (manganese), 8.2% (potassium), 8.5% (zinc), and 7.9% (iron) of the RDA per portion, respectively (Supplementary Table 7). In contrast to an earlier analysis, our analysis did not detect iron, copper, and manganese in milk (78). The cashew-based drink showed the higher values for selenium accounting for 3.7–10.3% of RDA versus 4.5–6.3% of RDA per portion for milk (Table 5 and Supplementary Table 7). Milk and cashew beverages may thus contribute to increasing dietary selenium intake in countries where soil selenium content is reduced such as Switzerland. As expected, milk represents a good source of iodine (82–150 μg kg^–1^) achieving 10.9 to 20.0% of RDA per portion (Table 5 and Supplementary Table 7). Milk is thus an interesting popularly positioned product that helps achieving adequate iodine status of the population. This becomes particularly relevant in countries with poor iodine resources such as Switzerland and for population groups exposed to higher risk of iodine deficiency such as pregnant and lactating women as well as young children (79). Significant levels of iodine were found in 1 soy (76.6 μg kg^–1^), 3 rice (21.9–39.4 μg kg^–1^), and 1 oat (43.9 μg kg^–1^) plant-based drinks, likely resulting from red algae addition that is known to increase iodine values (80, 81).

### Glyphosate, aminomethylphosphonic acid, and arsenic residues

All measured samples showed very low levels of glyphosate and AMPA (with the sum of glyphosate and AMPA < 2 ng mL^–1^), and therefore are well below the acceptable daily intake and acute reference dose (0.5 mg kg^–1^ of body weight per day) under conceivable consumption scenarios (82). However, if such chemical exposure doesn’t seem to raise safety concerns for adults, they might still be considered for specific groups such as infants and children. Rice drinks showed arsenic residues between 10.2 and 12.4 μg kg^–1^ and knowing that almost 80% of total arsenic is in its toxic inorganic form (iAs), we estimated that a consumption of three portions a day (0.6 L) of the tested rice drinks would correspond to an intake of approximately 6.5 μg of iAs (based on average arsenic concentration of 10.92 μg kg^–1^ in the five tested rice drinks) (83). This level of iAs exposure is not negligible as the mean intake for the Swiss population was recently estimated to be 0.029 μg kg^–1^ bw per day for adults and at 0.044 μg kg^–1^ bw for toddlers (1 to 3 years of age) (84). Although a benchmark dose lower confidence limit (BMDL01) as low as 0.3 μg kg^–1^ bw per day was defined, arsenic exposure should be kept as low as possible (85). It appears then that regular consumption of rice drinks could significantly contribute to arsenic exposure. One should also mention that these levels of arsenic were still within the range of the maximum value for drinking water that applies in Switzerland and the European Union (86, 87). Furthermore, the arsenic content of the red algae used for calcium fortification should also be monitored because of possible arsenic accumulations (88).

### Advantages, limits, and outlook for plant-based drinks

One portion (200 mL) of cow’s milk contributes for more than 10% of the RDA for biotin, pantothenic acid, vitamin B_2_, phosphorus, calcium and iodine (Supplementary Table 7). It can provide also a significant proportion of the daily requirement of proteins with high nutritional quality for humans. Another advantage for milk relies with the relative higher stability of its nutrient composition with the exception of iodine content that can vary depending on the season and feeding habits (89). Cow’s milk is also a good vector of fatty acids, with an interesting omega 6 to omega 3 ratio but still with a significant dominance of saturated fatty acids and a contribution to the intake of trans fatty acids. Plant-based drinks are clearly advantaged by their higher proportions of monounsaturated and polyunsaturated fatty acids except for coconut drink. Plant-based drinks and particularly soy-based drinks and products such as infant formulas are well indicated in cases of allergy and intolerance to cow’s milk proteins, hereditary lactase deficiency and galactosemia. However, the digestibility and nutritional performance of plant-based proteins is reduced by the natural occurrence of antinutrients and often limitations for specific essential amino acids (41). Whereas cow’s milk lactose is an issue in situation of lactose intolerance, soy is also known to bring raffinose and stachyose that can cause digestive discomfort. Phytic acid in plant-based products should also be taken into account as it can potentially limit the bioavailability of essential minerals even when such minerals are present in the products. Therefore, the anti-nutrient profile of plant-based products should be more frequently measured and communicated to consumers. Without fortification, plant-based drinks are limited in their ability to provide significant amounts of micronutrients unlike cow’s milk that is a richer source (e.g., vitamin B12, iodine) (90, 91). Amongst the tested plant-based drinks, soy-based drinks appear to have the closest nutritional characteristics to milk in terms of contributions to RDAs but still particular attention is needed before proceeding with total milk substitution. Moreover, it is worth mentioning that the long-term effect of regular intake of soy phytoestrogens idaidzein, genistein, glycitein), particularly in children, on endocrine functions and the reproductive system later in life requires more scientific evidence (41). In future, nutritional quality of plant-based drinks with science-proven nutrient and micronutrient bioavailability should be equally considered and communicated than sustainability goals (92). Practical solutions to mitigate nutritional gaps of specific plant-based drinks could be to opt, whenever possible, for a combination of plant-based alternatives as part of a balanced diet to ensure adequate fulfilment of nutrient and micronutrient needs. Finally, real innovation opportunities exist on evolving conventional and ultra-processing food manufacturing techniques, generally required in the manufacturing of plant-based products, toward simplified and/or natural processes such as microbial fermentation that can reduce antinutritional characteristics of foods/ingredients, improve protein digestibility and produce additional micro-or phytonutrients.

### Strength and limits

Our study shows a comprehensive laboratory analysis of the nutrient composition of plant-based products actually available in the Swiss Market. Not only the amount but also the quality of macronutrients was assessed. In addition, glyphosate and arsenic levels were determined to enable a risk assessment. We did not discriminate between enriched and non-enriched products to have a general overview.

However, this might be a limitation for the interpretation of some results. Furthermore, we did not analyze fiber and oligosaccharides, so the total amount of carbohydrates might be underestimated. For fiber we only added information from the literature. We did not detect the trace elements manganese and copper and discussed the possible reasons. Carotenoids, usually present in the form of vitamin A in plants, were not analyzed.

As plant-based drinks are a growing and highly volatile market, there are currently only a few different products available in each category. Statistical statements are therefore only possible to a limited extent.

## Conclusion

Our findings show that the analyzed plant-based beverages significantly differed in their nutrient composition, not only compared with cow’s milk but also between the drinks themselves. They were based on different food sources and provided different nutrient amounts and calories. Over years, the most important reasons for adding milk to our diet relies with its high nutrient density and quality, especially for protein and calcium. Only the soy-based beverages reached similar protein amounts but had lower protein quality, as assessed on the basis of the DIAAS. In addition, milk is a significantly richer dietary source of micronutrients such as calcium, iodine, vitamin B_2_, pantothenic acid, and biotin than plant-based drinks, which, by contrast, provide higher amounts of vitamin E and manganese, depending on the source. The measured nutrient values showed that the plant-based beverages cannot, as they stand, be considered nutritionally equivalent to cow’s milk and that their long-term consumption may require dietary adjustments to fully meet nutritional needs. In the case of fortified drinks, the question arises as to how the bioavailability of these added minerals and vitamins compares with milk. Studies on the digestibility and absorption of these drinks and their ingredients would provide further insights into their comparability with milk. Our results show that either the next generation of plant-based beverages must be optimized in nutrient profiles or combined with dietary adjustments if milk is to be fully substituted by plant-based drinks.

## Data availability statement

The original contributions presented in this study are included in the article/Supplementary material, further inquiries can be directed to the corresponding author.

## Author contributions

BW and KK-B created the conception and design of the study. RB, LE, RP, SD, MH, OZ, PR, and RV performed the analysis and measurements. BW, KK-B, and DG analyzed the data. DG carried out the statistics and produced the graphics. BW, KK-B, and SR wrote the manuscript. All authors read and approved the final manuscript.

## Footnotes

1
http://www.r-project.org
