# Supplementary material for: Comparison of nutritional composition between plant-based drinks and cow’s milk
Source: Front Nutr. 2022 Oct 28;9:988707. doi: 10.3389/fnut.2022.988707 (PMC9650290; doi:10.3389/fnut.2022.988707)
Supplement: Supplementary file 1 [file Image_1.pdf]

Figure S1: Variability of selected vitamins in the individual plant-based drink and cow's milk samples

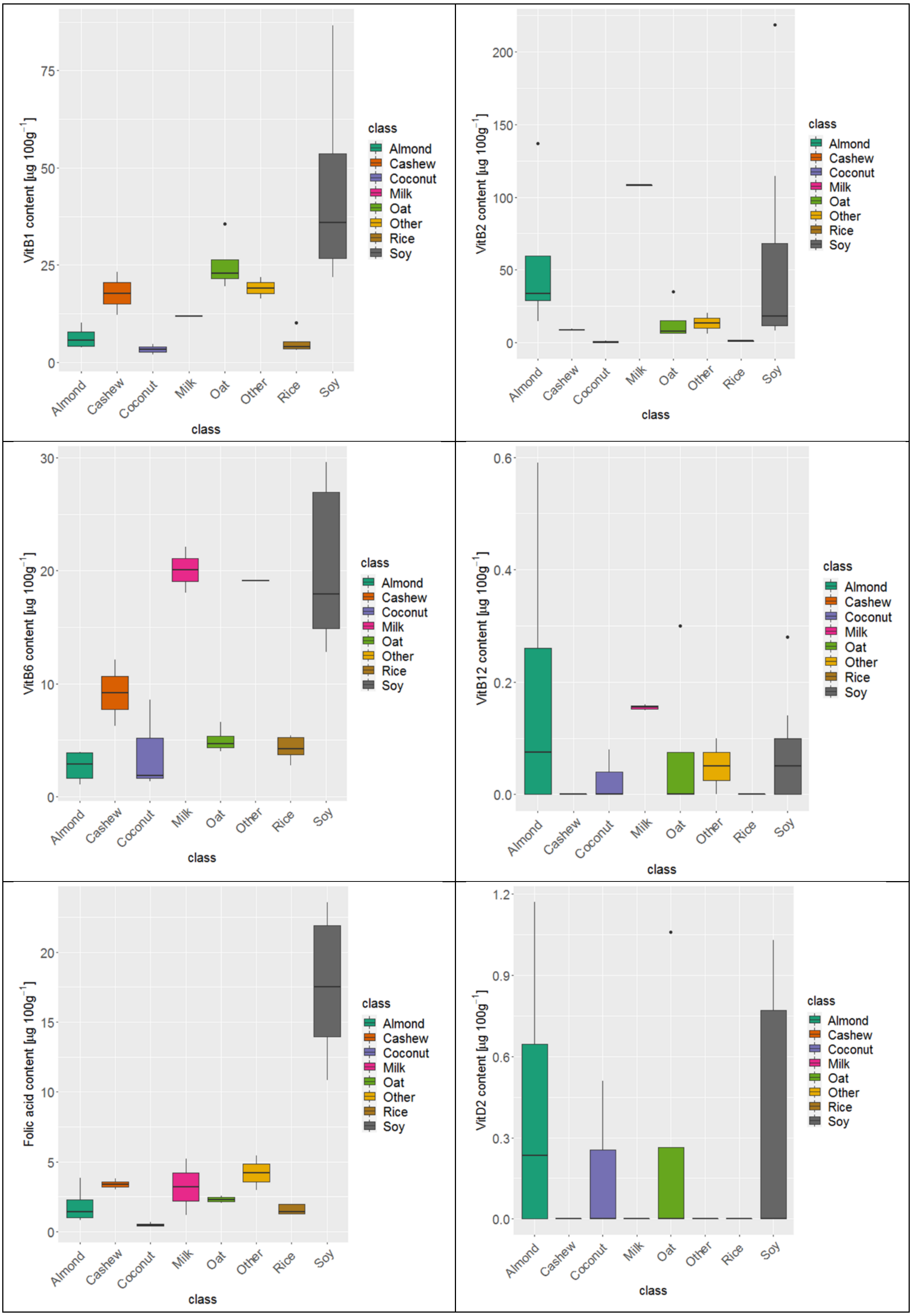

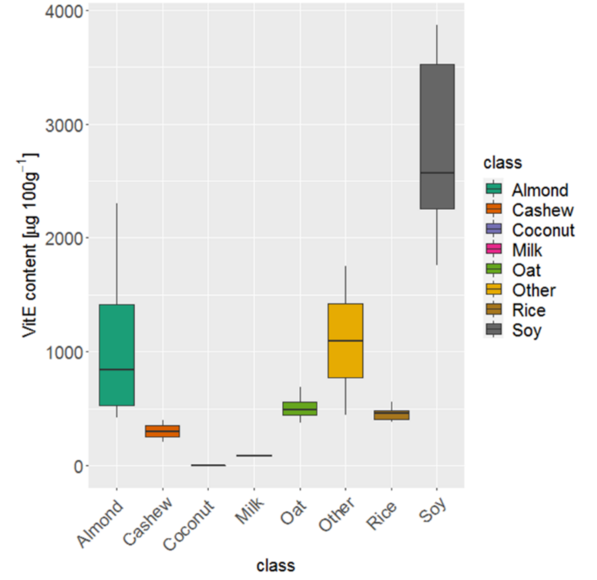
4000
3000
2000
1000
0
VitE content [µg 100g⁻¹]
Almond
Cashew
Coconut
Milk
Oat
Other
Rice
Soy
class
class
Almond
Cashew
Coconut
Milk
Oat
Other
Rice
Soy

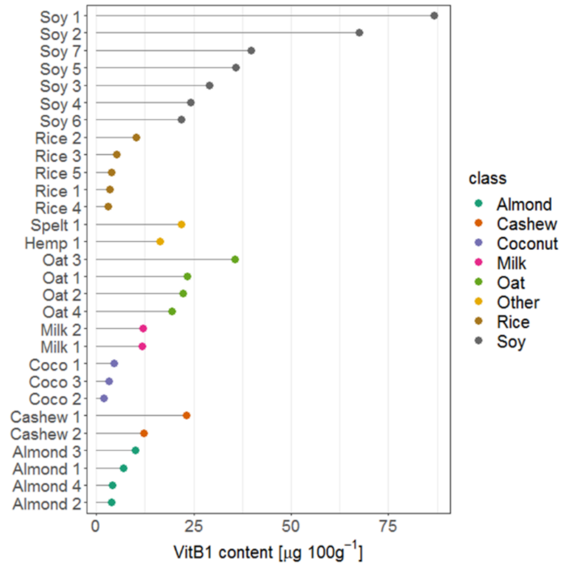
Soy 1
Soy 2
Soy 7
Soy 5
Soy 3
Soy 4
Soy 6
Rice 2
Rice 3
Rice 5
Rice 1
Rice 4
Spelt 1
Hemp 1
Oat 3
Oat 1
Oat 2
Oat 4
Milk 2
Milk 1
Coco 1
Coco 3
Coco 2
Cashew 1
Cashew 2
Almond 3
Almond 1
Almond 4
Almond 2
0
25
50
75
VitB1 content [µg 100g⁻¹]
class
Almond
Cashew
Coconut
Milk
Oat
Other
Rice
Soy

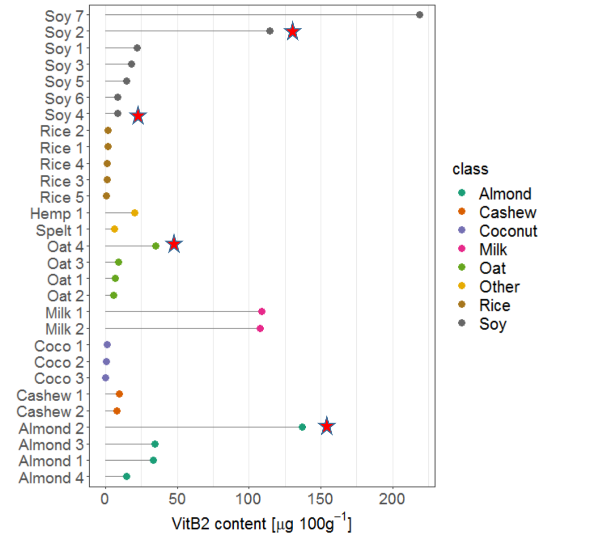
Soy 7
Soy 2
Soy 1
Soy 3
Soy 5
Soy 6
Soy 4
Rice 2
Rice 1
Rice 4
Rice 3
Rice 5
Hemp 1
Spelt 1
Oat 4
Oat 3
Oat 1
Oat 2
Milk 1
Milk 2
Coco 1
Coco 2
Coco 3
Cashew 1
Cashew 2
Almond 2
Almond 3
Almond 1
Almond 4
0
50
100
150
200
VitB2 content [µg 100g⁻¹]
class
Almond
Cashew
Coconut
Milk
Oat
Other
Rice
Soy

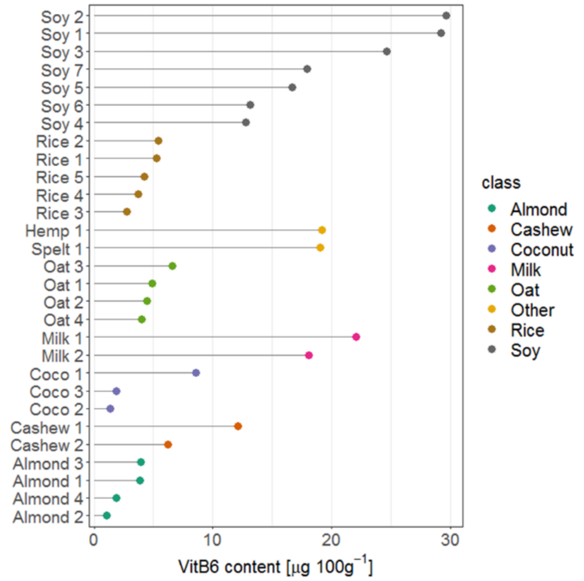
Soy 2
Soy 1
Soy 3
Soy 7
Soy 5
Soy 6
Soy 4
Rice 2
Rice 1
Rice 5
Rice 4
Rice 3
Hemp 1
Spelt 1
Oat 3
Oat 1
Oat 2
Oat 4
Milk 1
Milk 2
Coco 1
Coco 3
Coco 2
Cashew 1
Cashew 2
Almond 3
Almond 1
Almond 4
Almond 2
0
10
20
30
VitB6 content [µg 100g⁻¹]
class
Almond
Cashew
Coconut
Milk
Oat
Other
Rice
Soy

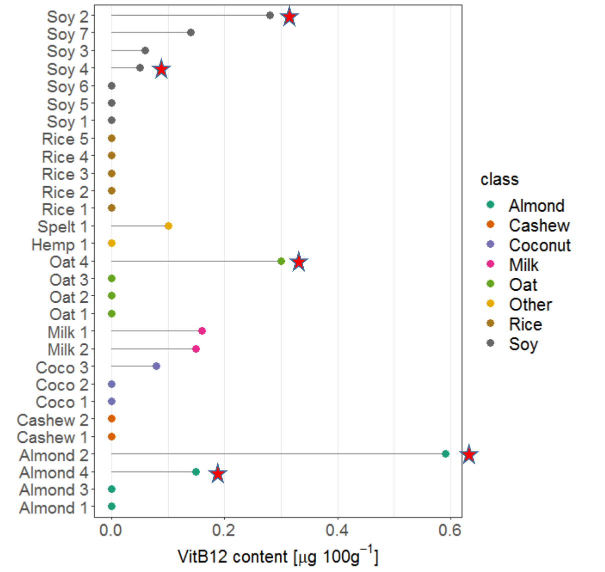
Soy 2
Soy 7
Soy 3
Soy 4
Soy 6
Soy 5
Soy 1
Rice 5
Rice 4
Rice 3
Rice 2
Rice 1
Spelt 1
Hemp 1
Oat 4
Oat 3
Oat 2
Oat 1
Milk 1
Milk 2
Coco 3
Coco 2
Coco 1
Cashew 2
Cashew 1
Almond 2
Almond 4
Almond 3
Almond 1
0.0
0.2
0.4
0.6
VitB12 content [µg 100g⁻¹]
class
Almond
Cashew
Coconut
Milk
Oat
Other
Rice
Soy

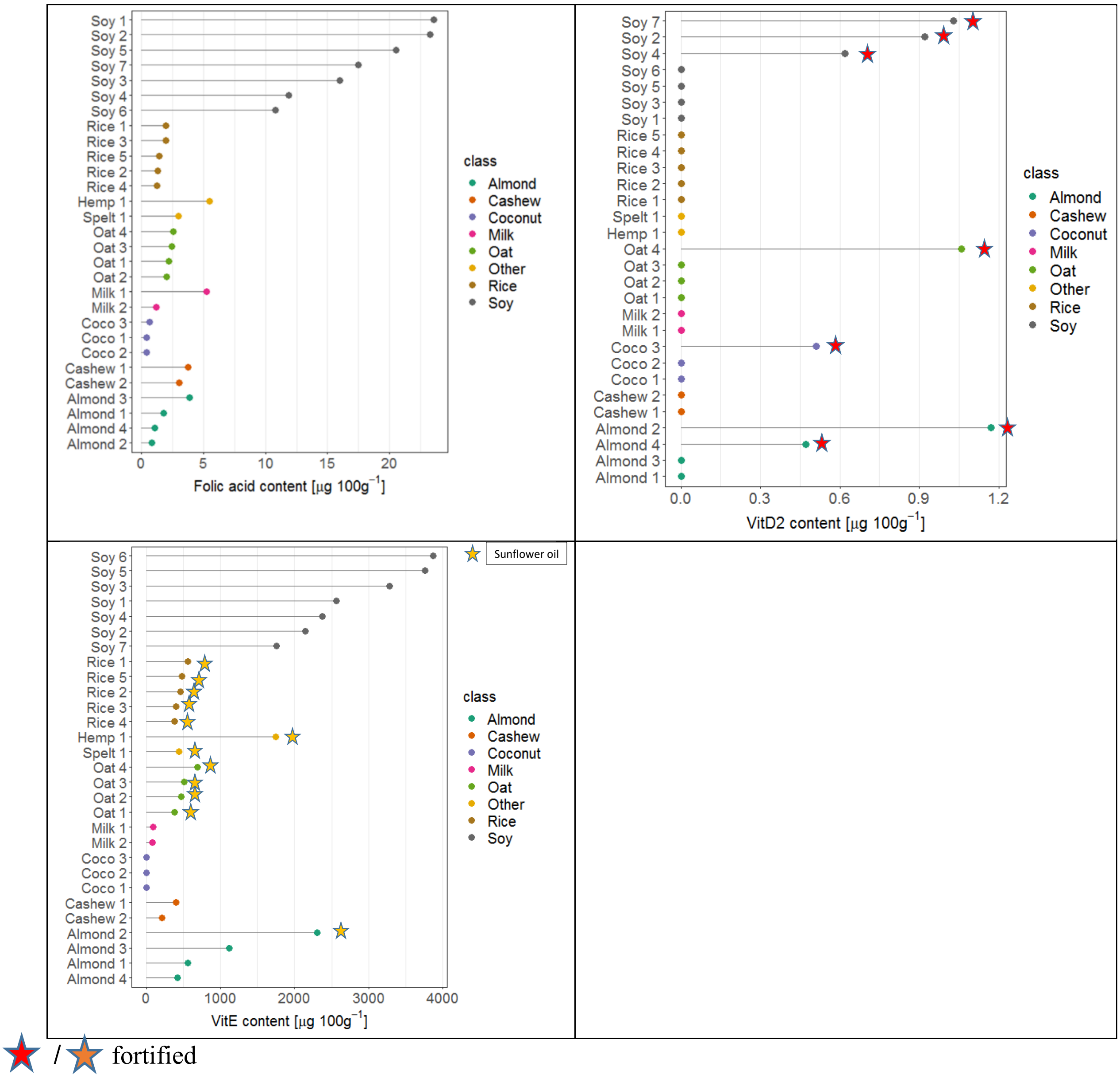

Soy 1
Soy 2
Soy 5
Soy 7
Soy 3
Soy 4
Soy 6
Rice 1
Rice 3
Rice 5
Rice 2
Rice 4
Hemp 1
Spelt 1
Oat 4
Oat 3
Oat 1
Oat 2
Milk 1
Milk 2
Coco 3
Coco 1
Coco 2
Cashew 1
Cashew 2
Almond 3
Almond 1
Almond 4
Almond 2
0 5 10 15 20
Folic acid content [µg 100g^{-1}]
class
Almond
Cashew
Coconut
Milk
Oat
Other
Rice
Soy
Soy 7
Soy 2
Soy 4
Soy 6
Soy 5
Soy 3
Soy 1
Rice 5
Rice 4
Rice 3
Rice 2
Rice 1
Spelt 1
Hemp 1
Oat 4
Oat 3
Oat 2
Oat 1
Milk 2
Milk 1
Coco 3
Coco 2
Coco 1
Cashew 2
Cashew 1
Almond 2
Almond 4
Almond 3
Almond 1
0.0 0.3 0.6 0.9 1.2
VitD2 content [µg 100g^{-1}]
class
Almond
Cashew
Coconut
Milk
Oat
Other
Rice
Soy
Sunflower oil
Soy 6
Soy 5
Soy 3
Soy 1
Soy 4
Soy 2
Soy 7
Rice 1
Rice 5
Rice 2
Rice 3
Rice 4
Hemp 1
Spelt 1
Oat 4
Oat 3
Oat 2
Oat 1
Milk 1
Milk 2
Coco 3
Coco 2
Coco 1
Cashew 1
Cashew 2
Almond 2
Almond 3
Almond 1
Almond 4
0 1000 2000 3000 4000
VitE content [µg 100g^{-1}]
class
Almond
Cashew
Coconut
Milk
Oat
Other
Rice
Soy
/ fortified
