# Supplementary material for: Comparison of nutritional composition between plant-based drinks and cow’s milk
Source: Front Nutr. 2022 Oct 28;9:988707. doi: 10.3389/fnut.2022.988707 (PMC9650290; doi:10.3389/fnut.2022.988707)
Supplement: Supplementary file 2 [file Image_2.pdf]

Figure S2: Variability of selected minerals in individual plant-based drink and cow's milk samples

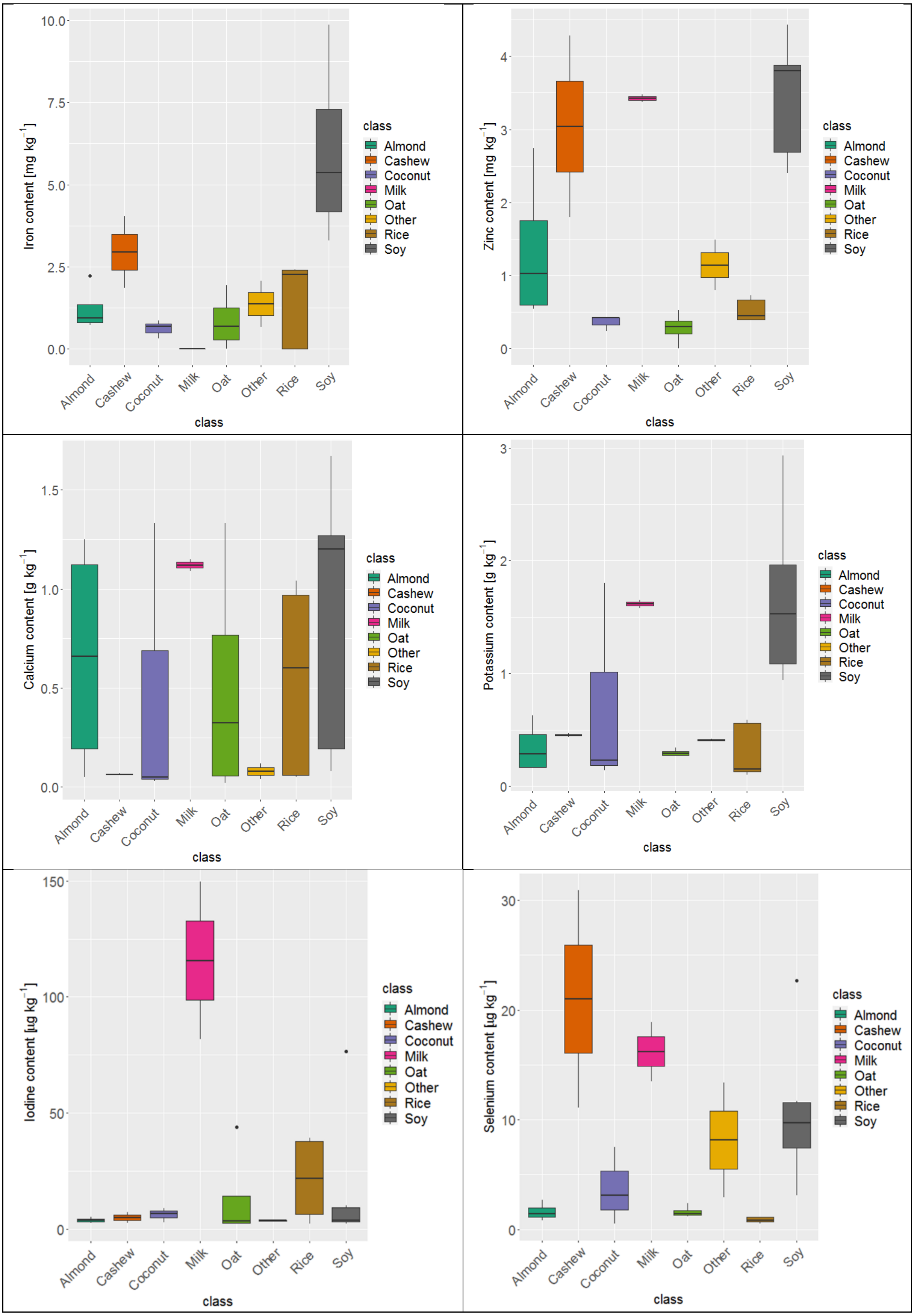

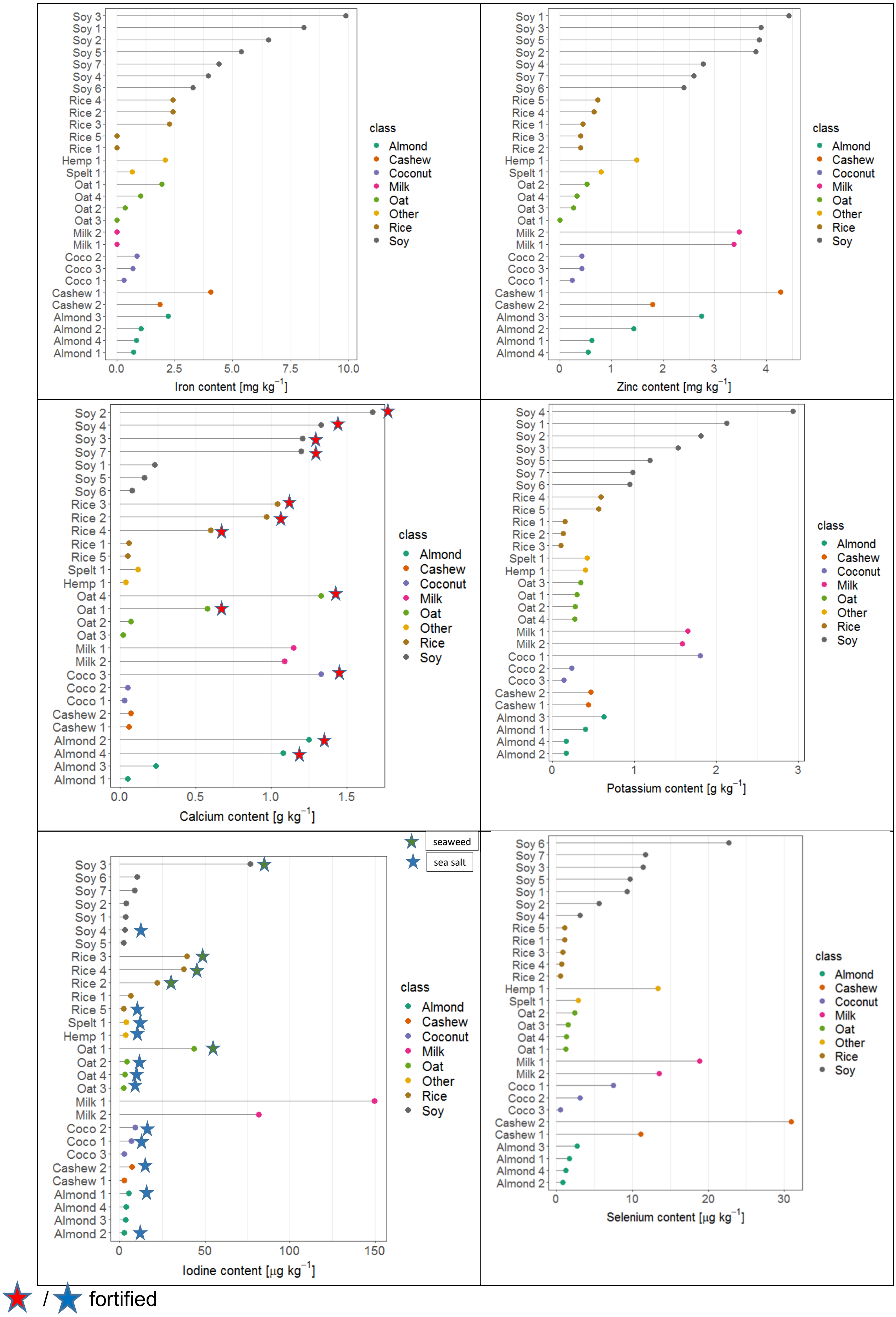
Iron content [mg kg^{-1}]
Zinc content [mg kg^{-1}]
Calcium content [g kg^{-1}]
Potassium content [g kg^{-1}]
Iodine content [µg kg^{-1}]
Selenium content [µg kg^{-1}]
class
Almond
Cashew
Coconut
Milk
Oat
Other
Rice
Soy
seaweed
sea salt
/ fortified
