# Supplementary material for: Comparison of nutritional composition between plant-based drinks and cow’s milk
Source: Front Nutr. 2022 Oct 28;9:988707. doi: 10.3389/fnut.2022.988707 (PMC9650290; doi:10.3389/fnut.2022.988707)
Supplement: Supplementary file 3 [file Table_1.pdf]

Table S1. List of products and their ingredients according to the label

Sample	Ingredients
Almond 1	Water, ALMONDS 7%, sea salt.
Almond 2	Water, sugar, ALMONDS (2.3%), tricalcium phosphate, sea salt, stabilizers (carob gum, gellan), emulsifier (sunflower lecithin), vitamins (riboflavin (B2) (0.21mg/100ml), B12 (0.38 μg/100ml), E (1.8mg/ml), D2 (0.75 μg/100ml).
Almond 3	Water, ALMOND PASTE 8%, raw cane sugar, cooking salt.
Almond 4	Water, ALMOND (2%), mineral salt: calcium phosphate, sodium chloride, stabilizers: gellan (SOY) and locust bean gum, emulsifier: sunflower lecithin, acidity regulator: sodium bicarbonate, natural flavorings, sweeteners: steviol glycosides, vitamins: D (0.75 μg/100ml) and B12 (0.38 μg/100ml).
Cashew 1	Water, CASHEWPASTE 6%, rice flour 3%, sea salt.
Cashew 2	Water, CASHEWKERNELS 6.5%, sea salt.
Coconut 1	Water, coconut extract 8%, sea salt.
Coconut 2	Water, coconut extract 6.6%, raw cane sugar, natural flavoring, sea salt, thickening agent: E 407, stabilizer: E 418. Total content 98.2% without water, of which sugar types with quantity compensation.
Coconut 3	Water, coconut milk (5.3%) (coconut cream, water), rice (3.3%), tricalcium phosphate, stabilizers (carrageenan, guar gum, xanthan), sea salt, vitamins (B12 (0.38 μg/100ml), D2 (0.75 μg/100ml)), flavorings.
Hemp 1	Water, hemp flour 5%, sunflower oil, cornstarch, sea salt.
Milk 1	Bovine milk, UHT 3.5% fat.
Milk 2	Bovine milk, UHT 3.5% fat.
Oat 1	Water, whole OATS 11%, sunflower oil, sea-red alga 0.4%, sea salt.
Oat 2	Water, OATS WHOLE 11%, sunflower oil, sea salt.
Oat 3	Water, OAT 11%, sunflower oil, sea salt.
Oat 4	Water, OAT (10%), inulin, sunflower oil, tricalcium orthophosphate, maltodextrin, sea salt, stabilizer (gellan gum), vitamins (riboflavin (B2) (0.21mg/100ml), B12 (0.38 μg/100ml), D2 (0.75 μg/100ml)).
Rice 1	Water, rice powder 8.5% (rice syrup, rice starch, rice flour), sunflower oil, rice starch, cane sugar raw, rice maltodextrin, sunflower lecithin, thickener: E 407.
Rice 2	Water, rice 14%, sunflower oil, calcium-containing red algae (Lithothamnium calcareum) 0.4%, sea salt.
Rice 3	Water, rice 14%, sunflower oil, marine red alga (Lithothamnium calcareum) 0.4%, sea salt.
Rice 4	Water, rice flour 14% (Italy), sunflower oil, calcium-containing red algae powder (Lithothamnium calcareum), sea salt.
Rice 5	Water, rice flour 14%, sunflower oil, sea salt.
Soy 1	Water, soybeans 8.5%
Soy 2	Water, soybeans 7.2%, sugar, calcium phosphate, table salt, vitamin B2 (0.1mg/100ml), vitamin D (0.4μg/100ml) and vitamin B12 (0.2 μg/100ml)
Soy 3	Water, SOYBEANS 8%, cane sugar, seaweed Lithothamnium 0.4%, sea salt.
Soy 4	Water, shelled SOYBEANS (5.9%), sugar, tricalcium phosphate, acidity regulator (monopotassium phosphate), sea salt, flavor, stabilizer (gellan), vitamins (riboflavin (B2) (0.21mg/100ml), B12 (0.38 μg/100ml), D2 (0.75 μg/100ml)).
Soy 5	Water, Organic SOYBEANS 9%.
Soy 6	SOYA DRINK 100%
Soy 7	SOYDRINK 97% (water, SOYBEANS 7%), sugar, calcium phosphate, stabilizer: gellan, natural flavor, salt, vitamin D (0.75 μg/100ml).
Spelt 1	Water, spelt flour (11%), sunflower oil, sea salt.
