# Supplementary material for: Comparison of nutritional composition between plant-based drinks and cow’s milk
Source: Front Nutr. 2022 Oct 28;9:988707. doi: 10.3389/fnut.2022.988707 (PMC9650290; doi:10.3389/fnut.2022.988707)
Supplement: Supplementary file 4 [file Table_2.pdf]

Table S2: List of analytical methods used for the determination of vitamins in the different plant-based drinks and cow's milk

Vitamins	Standards	Reference	Preparation	Analysis	LOD	LOQ
B1	Thiamine hydrochloride Sigma T 4625	Mai H. Bui, Int. J. Vitam. Nutr. Res., 69 (4), 1999	Hot acid hydrolysis	Microbiological assay: *Lactobacillus fermentis*; turbidimetry	0,2 ng mL^{-1}	0,67 ng mL^{-1}
B2	Riboflavin Sigma R4500	AOAC 940.33	Hot acid hydrolysis	Microbiological assay: *Lactobacillus casei*; turbidimetry	1,1 ng mL^{-1}	3,6 ng mL^{-1}
B6	B6 hydrochloride Fluka 95180	AOAC 961.15	Hot acid hydrolysis	Microbiological assay: *Saccharomices carlsbergensis*; turbidimetry	0,08 ng mL^{-1}	0,28 ng mL^{-1}
Niacin	Nicotinamide Sigma 72340	AOAC 944.13	Hot acid hydrolysis	Microbiological assay: *Lactobacillus plantarum*; turbidimetry	1,9 ng mL^{-1}	6 ng mL^{-1}
Pantothenic acid	D-Pantothenic acid hemicalcium salt Sigma 21210	AOAC 945.74	Hot water extraction	Microbiological assay: *Lactobacillus plantarum*; turbidimetry	3,8 ng mL^{-1}	11 ng mL^{-1}
Biotin	Biotine Sigma B4501	Schw. LMB 1550.1	Hot acid hydrolysis	Microbiological assay: *Lactobacillus plantarum*; turbidimetry	0,02 ng mL^{-1}	0,07 ng mL^{-1}
Folic acid	Folic acid Sigma F7876	AOAC 2004.05 / EN 14131: 2003, Or DEVRIES ET AL.: JOURNAL OF AOAC INTERNATIONAL VOL. 88, NO. 1, 2005	Hot basic or trienzyme extraction	Microbiological assay: *Lactobacillus casei*; turbidimetry	0,01 ng mL^{-1}	0,03 ng mL^{-1}
B12	Cyanocobalamin Sigma PHR1234	AOAC 952.20	Hot water extraction (buffered)	Microbiological assay: *Lactobacillus leichmanii*; turbidimetry	0.0017 ng mL^{-1}	0.0056 ng mL^{-1}
C	Acide-L-Ascorbique Sigma 33034	EN 14130:2003 / M.H. Bui-Nguyên, J. Chromatogr. 196,163, 1980	Extraction with acetic or metaphosphoric acid	HPLC; UV detection	24 ng mL^{-1}	80 ng mL^{-1}
A	A alcohol Fluka 95144	AOAC 2001.13	Saponification, extraction	HPLC; UV detection	40 ng mL^{-1}	130 ng mL^{-1}
E	DL-α-Tocophérol Fluka 95240	AOAC 992.03	Saponification, extraction	HPLC; UV detection	300 ng mL^{-1}	1100 ng mL^{-1}

D	D3 Fluka 95230/D2 Fluka 95220<br>D2 Lanospharma Laboratories Co.,Ltd<br>D4 Lanospharma Laboratories Co.,Ltd	AOAC 2002.05	Saponification, extraction	HPLC; UV detection, or LC/MS	35 ng mL^{-1}	120 ng mL^{-1}
K1<br>K2	Phylloquinone Sigma 47773<br>Menaquinone Sigma 4774	Schw. LMB 1540.1	Enzymatic digestion, liquid/liquid extraction	HPLC; fluorimetric detection	0,015 ng mL^{-1}	0,06 ng mL^{-1}
