# Supplementary material for: Comparison of nutritional composition between plant-based drinks and cow’s milk
Source: Front Nutr. 2022 Oct 28;9:988707. doi: 10.3389/fnut.2022.988707 (PMC9650290; doi:10.3389/fnut.2022.988707)
Supplement: Supplementary file 6 [file Table_4.pdf]

Table S4. Calculated mean DIAAS and predicted first-limiting amino acids for the different plant-based drinks and cow's milk for different reference patterns.

Product		Reference Pattern					
		Infants (birth to 6 months)		Children (6 months to 3 years)		Older children, adolescents and adults	
	N	DIAAS (%)	1st Lim eAA	DIAAS (%)	1st Lim eAA	DIAAS (%)	1st Lim eAA
Almond Drink	4	27.1	Lys	32.9	Lys	39.0	Lys
Cashew Drink	2	51.2	Ile	66.4	Lys	78.8	Lys
Coconut Drink	3	39.1	TRP	62.8	Lys	71.9	Ile
Cow's milk	2	65.4	TRP	123.5	SAA	145.0	SAA
Hemp Drink	1	38.4	Lys	46.5	Lys	55.2	Lys
Oat Drink	4	41.1	Lys	49.8	Lys	59.1	Lys
Rice Drink	5	30.0	Lys	36.3	Lys	43.1	Lys
Soy Drink	7	71.2	TRP	91.9	SAA	107.6	Val
Spelt Drink	1	28.5	Lys	34.5	Lys	41.0	Lys

DIAAS: Digestible Indispensable Amino Acid Score, 1st Lim eAA: first limiting essential amino acid, Ile: isoleucine, Lys: lysin, Leu: leucin, TRP: Tryptophan,Val: valin, SAA: sulphur amino acid
