# Supplementary material for: Comparison of nutritional composition between plant-based drinks and cow’s milk
Source: Front Nutr. 2022 Oct 28;9:988707. doi: 10.3389/fnut.2022.988707 (PMC9650290; doi:10.3389/fnut.2022.988707)
Supplement: Supplementary file 8 [file Table_6.pdf]

Table S6. Concentrations of glyphosate, aminomethylphosphonic acid (AMPA), and arsenic in the different plant-based drinks and cow's milk

	Glyphosate	AMPA	Arsenic
	Ng mL^{-1}	Ng mL^{-1}	µg kg^{-1}
Soy 1	Traces < 0.3	0.7	2.6
Soy 2	0.8	0.6	3.4
Soy 3	nd < 0.1	Traces < 0.3	4.3
Soy 4	nd < 0.1	Traces < 0.3	3.3
Soy 5	nd < 0.1	0.7	2.8
Soy 6	nd < 0.1	Traces < 0.3	2.4
Soy 7	nd < 0.1	Traces < 0.3	2.6
Almond 1	Traces < 0.3	Traces < 0.3	2.7
Almond 2	Traces < 0.3	Traces < 0.3	3.3
Almond 3	Traces < 0.3	0.4	2.5
Almond 4	0.3	Traces < 0.3	3.2
Rice 1	Traces < 0.3	Traces < 0.3	10.7
Rice 2	Traces < 0.3	Traces < 0.3	11.2
Rice 3	Traces < 0.3	Traces < 0.3	10.1
Rice 4	Traces < 0.3	Traces < 0.3	12.4
Rice 5	Traces < 0.3	0.3	10.2
Coconut 1	nd < 0.1	nd < 0.1	2.0
Coconut 2	nd < 0.1	Traces < 0.3	2.2
Coconut 3	Traces < 0.3	Traces < 0.3	4.6
Oat 1	nd < 0.1	nd < 0.1	3.6
Oat 2	nd < 0.1	Traces < 0.3	3.7
Oat 3	Traces < 0.3	Traces < 0.3	2.3
Oat 4	0.7	Traces < 0.3	3.6
Cashew 1	Traces < 0.3	Traces < 0.3	4.4
Cashew 2	nd < 0.1	nd < 0.1	2.5
Hemp	0.6	Traces < 0.3	3.0
Spelt	nd < 0.1	nd < 0.1	2.4
Cow's milk 1	nd < 0.1	nd < 0.1	3.9
Cow's milk 2	nd < 0.1	nd < 0.1	3.3

nd: not detected, limit of determination (LOD): 0.1 ng/ml, limit of quantification (LOQ): 0.3 ng/ml
