# Supplementary material for: Comparison of nutritional composition between plant-based drinks and cow’s milk
Source: Front Nutr. 2022 Oct 28;9:988707. doi: 10.3389/fnut.2022.988707 (PMC9650290; doi:10.3389/fnut.2022.988707)
Supplement: Supplementary file 9 [file Table_7.pdf]

Table S7. Mean % RDA and ranges of the macro- and micro-nutrient concentrations and energy per portion (200 mL) for various plant-based drinks and cow's milk consumed by adult women (aged 19–65 years)

	Almond Drink	Cashew Drink	Coconut Drink	Cow's Milk	Hemp Drink	Oat Drink	Rice Drink	Soy Drink	Spelt Drink
n	4	2	3	2	1	4	5	7	1
Vitamin C	-	-	-	0.5 (0.2–0.9)	-	-	-	-	-
Biotin	3.7 (2.4–5.6)	4.4 (2.8–6.1)	5.0 (1.0–11.3)	11.5 (9.8–13.3)	9.5	8.5 (7.5–10.9)	1.9 (1.3–2.8)	9.4 (5.2–15.0)	3.8
Niacin	2.7 (1.4–4.5)	1.4 (1.1–1.6)	1.7 (0.4–3.5)	2.2 (2.1–2.3)	3.7	1.0 (0.8–1.4)	2.2 (1.9–2.6)	2.9 (1.5–4.7)	3.8
Pantothenic acid	0.6 (0.2–1.1)	3.1 (2.7–3.6)	1.0 (0.5–1.6)	11.9 (11.0–12.9)	5.1	4.9 (4.5–5.2)	4.4 (3.2–6.3)	3.9 (2.5–6.4)	3.0
Vitamin B1	1.3 (0.8–2.0)	3.5 (2.4–4.6)	0.7 (0.4–0.9)	2.4 (2.4)	3.3	5.0 (3.9–7.1)	1.0 (0.6–2.0)	8.7 (4.4–17.3)	4.4
Vitamin B2	10.0 (2.7–24.9)[1)]	1.6 (1.5–1.7)	0.1 (0–0.3)	19.7 (19.6–19.8)	3.7	2.6 (1.1–6.3)[1)]	0.2 (0.2–0.3)	10.5 (1.5–39.7)[1)]	1.1
Vitamin B6	0.4 (0.2–0.6)	1.3 (0.9–1.7)	0.6 (0.2–1.2)	2.9 (2.6–3.2)	2.7	0.7 (0.6–0.9)	0.6 (0.4–0.8)	2.9 (1.8–4.2)	2.7
Vitamin B12	9.3 (0–29.6) [1)]	-	1.4 (0–4.2) [1)]	7.8 (7.6–8.1)	-	3.8 (0–15.2)[1)]	-	3.8 (0–13.8)[1)]	5.0
Folic acid	2.5 (1.1–5.2)	4.5 (4.0–5.0)	0.7 (0.5–0.9)	4.3 (1.6–7.0)	7.3	3.1 (2.7–3.4)	2.1 (1.6–2.6)	23.5 (14.4–31.4)	3.9
Vitamin A	-	-	-	5.8 (5.5–6.2)	-	-	-	-	-
Vitamin E	18.4 (7.0–38.4)[1)]	5.1 (3.5–6.6)	-	1.5 (1.4–1.6)	29.2[1)]	8.6 (6.3–11.6)[1)]	7.6 (6.4–9.3)[1)]	47.0 (29.3–64.4)	7.4[1)]
Vitamin D2	5.5 (0–15.6) [1)]	-	2.3 (0–6.9) [1)]	-	-	3.5 (0–14.1) [1)]	-	4.9 (0–13.7) [1)]	-
Vitamin K (K_1 + K_2)	-	6.0 (3.4–8.6)	-	1.9 (1.9)	10.6	0.4 (0.3–0.4)	0.0 (0–0.1)	11.5 (7.1–15.9)	0.4
P	12.4 (3.1–18.7)	9.6 (6.5–12.8)	8.5 (1.5–18.9)	26.4 (24.9–27.9)	7.6	8.3 (3.1–20.9)	2.0 (1–3.8)	23.1 (13.1–37.0)	8.9
Na	7.0 (3.3–11.4)	4.1 (2.5–5.7)	4.5 (3.4–5.9)	5.1 (4.9–5.2)	7.6	5.3 (4.1–6.1)	2.0 (0.4–4.3)	3.1 (0.1–5.7)	5.9
Mn	2.3 (1.1–4.6)	6.0 (2.5–9.5)	2.0 (1.7–2.3)	-	7.0	0.9 (0–1.8)	0.5 (0–2.3)	12.3 (7.8–16.4)	2.7
Mg	6.3 (4.2–11.6)	10.6 (7.4–13.7)	3.9 (2.1–6.3)	6.7 (6.5–6.8)	5.1	2.8 (1.3–4.7)	4.5 (2.1–6.5)	13.4 (8.7–18.2)	4.8
K	1.7 (0.8–3.2)	2.3 (2.2–2.4)	3.6 (0.7–9.0)	8.1 (7.9–8.3)	2.0	1.5 (1.3–1.7)	1.5 (0.5–3.0)	8.2 (4.7–14.7)	2.1
Fe	1.6 (1.0–3.0)	3.9 (2.5–5.4)	0.8 (0.4–1.2)	-	2.8	1.1 (0–2.6)	1.9 (0–3.2)	7.9 (4.4–13.2)	0.9
Cu	7.6 (3.4–14.8)	20.6 (15.7–25.5)	4.1 (0–9.2)	-	13.0	1.1 (0–4.3)	-	21.3 (16.3–27.4)	4.4
Ca	13.1 (1.1–25.0)[1)]	1.3 (1.2–1.4)	9.4 (0.7–26.6)[1)]	22.4 (21.9–23)	0.9	10.0 (0.3–26.7)[1)]	10.9 (1.0–20.8)[1)]	16.8 (1.7–33.5)[1)]	2.4
Zn	3.3 (1.4–6.9)	7.6 (4.5–10.7)	0.9 (0.6–1.1)	8.6 (8.4–8.7)	3.7	0.7 (0–1.3)	1.3 (1.0–1.8)	8.5 (6.0–11.1)	2.0
Se	0.5 (0.3–0.9)	7.0 (3.7–10.3)	1.2 (0.2–2.5)	5.4 (4.5–6.3)	4.5	0.5 (0.4–0.8)	0.3 (0.2–0.4)	3.5 (1.0–7.6)	1.0
I	0.5 (0.3–0.7)	0.7 (0.4–1.0)	0.8 (0.4–1.2)	15.4 (10.9–20.0)	0.5	1.7 (0–5.9)	2.8 (0–5.3)	2 (0–10.2)	0.5
Cl	6.0 (3.5–7.8)	3.0 (0.5–5.5)	5.9 (4.0–7.4)	8.5 (8.5)	6.5	5.6 (4.8–6.0)	5.0 (1.2–8.3)	1.0 (0–3.7)	6.0

Protein	3.8 (2.0–6.8)	5.0 (4.5–5.4)	1.2 (0.4–2.3)	13.6 (13.6)	2.7	1.7 (1.0–2.2)	0.7 (0.3–1.1)	14.1 (11.7–17.9)	2.5
Carbohydrates	1.0 (0.1–1.9)	1.0 (0.8–1.2)	1.5 (0.8–2.7)	4.1 (4.1)	0.7	3.1 (0.3–5.8)	2.4 (0.5–3.2)	2.8 (0.4–6.5)	3.9
Fat	8.2 (3.5–15.9)	8.8 (8.3–9.4)	3.3 (0.4–6.3)	11.3 (11.2–11.5)	10.4	4.7 (4.2–4.9)	4.0 (3.1–7.0)	6.6 (5.2–9.0)	3.9
Energy*	3.6 (1.4–6.7)	4.6 (3.8–5.4)	2.6 (1.3–4.1)	7.3 (7.1–7.2)	3.4	4.6 (4.3–5.0)	6.1 (5.3–7.6)	4.6 (3.9–5.8)	4.7
Mean of % RDA per portion	4.6	4.4	2.3	7.5	5.4	3.3	2.1	10.4	3.1

*Female (20-50 years, PAL 1,4, 1800kcal/day; [1)] contains products with supplementation; in grey highest RDA for the specific nutrient.

-: concentration of nutrients was under detection limit, so no value for % RDA was calculated.
